# Children’s Phthalate Intakes and Resultant Cumulative Exposures Estimated from Urine Compared with Estimates from Dust Ingestion, Inhalation and Dermal Absorption in Their Homes and Daycare Centers

**DOI:** 10.1371/journal.pone.0062442

**Published:** 2013-04-23

**Authors:** Gabriel Bekö, Charles J. Weschler, Sarka Langer, Michael Callesen, Jørn Toftum, Geo Clausen

**Affiliations:** 1 International Centre for Indoor Environment and Energy, Department of Civil Engineering, Technical University of Denmark, Lyngby, Denmark; 2 Environmental and Occupational Health Sciences Institute, University of Medicine and Dentistry of New Jersey and Rutgers University, Piscataway, New Jersey, United States of America; 3 Swedish Environmental Research Institute, Göteborg, Sweden; 4 Pediatric Research Unit, Hans Christian Andersen Children’s Hospital, Odense University Hospital, Odense, Denmark; Stony Brook University, Graduate Program in Public Health, United States of America

## Abstract

Total daily intakes of diethyl phthalate (DEP), di(n-butyl) phthalate (DnBP), di(isobutyl) phthalate (DiBP), butyl benzyl phthalate (BBzP) and di(2-ethylhexyl) phthalate (DEHP) were calculated from phthalate metabolite levels measured in the urine of 431 Danish children between 3 and 6 years of age. For each child the intake attributable to exposures in the indoor environment via dust ingestion, inhalation and dermal absorption were estimated from the phthalate levels in the dust collected from the child’s home and daycare center. Based on the urine samples, DEHP had the highest total daily intake (median: 4.42 µg/d/kg-bw) and BBzP the lowest (median: 0.49 µg/d/kg-bw). For DEP, DnBP and DiBP, exposures to air and dust in the indoor environment accounted for approximately 100%, 15% and 50% of the total intake, respectively, with dermal absorption from the gas-phase being the major exposure pathway. More than 90% of the total intake of BBzP and DEHP came from sources other than indoor air and dust. Daily intake of DnBP and DiBP from all exposure pathways, based on levels of metabolites in urine samples, exceeded the Tolerable Daily Intake (TDI) for 22 and 23 children, respectively. Indoor exposures resulted in an average daily DiBP intake that exceeded the TDI for 14 children. Using the concept of relative cumulative Tolerable Daily Intake (TDI_cum_), which is applicable for phthalates that have established TDIs based on the same health endpoint, we examined the cumulative total exposure to DnBP, DiBP and DEHP from all pathways; it exceeded the tolerable levels for 30% of the children. From the three indoor pathways alone, several children had a cumulative intake that exceeded TDI_cum_. Exposures to phthalates present in the air and dust indoors meaningfully contribute to a child’s total intake of certain phthalates. Such exposures, by themselves, may lead to intakes exceeding current limit values.

## Introduction

Phthalates are a group of ubiquitous chemicals present in many consumer products, including building materials, furnishings, clothing, paints, food packaging, toys, personal care products and pharmaceuticals. Many of them are or have been produced in very large quantities. Phthalates can be released into the environment by leaching, evaporation, migration, abrasion or application of phthalate-containing personal care products. Due to their widespread use, the general population is continuously exposed to phthalates.

A large number of human and animal studies have focused on possible health effects of phthalate exposure. Articles reviewing the available literature are frequently published, and we refer to them for further details on the health effects of phthalate exposure. Briefly, phthalates are known to be developmental and reproductive toxicants. Indications exist that they may impact genital development, semen quality, children’s neurodevelopment, thyroid function, onset of puberty in females and that they may possibly cause respiratory problems [1],[2],[3],[4],[5],[6],[7],[8],[9]. Recent studies indicate that prenatal phthalate exposure may influence a child’s mental, psychomotor and behavioral development [10],[11],[12],[13], sex hormone status in newborns [14] and the risk of developing eczema in early childhood [15]. DEHP exposure of fertile men can be associated with minor alterations of markers of free testosterone [16]. DEHP metabolites were found at increased levels in children with autism [17] and school children with lower vocabulary and IQ scores [18]. Additionally, Toft et al. [19] found an association between periconceptional urinary concentration of MEHP, the primary metabolite of DEHP, and pregnancy loss. Exposure to certain phthalates may be associated with delayed pubarche in girls [20], obesity [21], biomarkers for inflammation and oxidative stress [22] and the genesis of diabetes [23]. Furthermore, there is some evidence that the secondary oxidized metabolites of DEHP may themselves be developmental toxicants [24],[25].

In the European Union the use of several phthalates in toys, child care articles and personal care products has been restricted based on either their classification as reproductive/developmental toxicants or the precautionary principle [26]. In February 2011 the European Parliament decided to phase out the use of DnBP, BBzP and DEHP by August 2015 (Annex XIV to Regulation No. 1907/2006 for Registration, Evaluation, Authorization and Restriction of Chemicals - REACH). Moreover, various authorities have established limit values for the intake of certain phthalates [27]. Limit values that are most often used are the Reference Dose (RfD) established by the US EPA and the Tolerable Daily Intake (TDI) established by the European Food Safety Authority (EFSA). Separate values have been established for different phthalates (see Table 1). Recent animal studies suggest that phthalates and other potential endocrine disrupting chemicals can act in a dose-additive manner [28],[29],[30],[31],[32],[33],[34],[35], [36]. The development of a method to evaluate the cumulative exposure to anti-androgenic chemicals has been advocated for some time [37],[38]. One such approach for assessing cumulative risk of phthalate exposure, the relative cumulative Tolerable Daily Intake (TDI_cum_), has been introduced by Koch et al. [26] in analogy to the Hazard Index (HI) [39],[38] and was recently reported by Søeborg et al. [40] for 129 Danish children and adolescents.

**Table 1:** Properties of the target phthalates and their metabolites used for the estimation of phthalate intake from the concentration in urine and from phthalate exposures in the indoor environment.

Parent phthalate	MW_1_ (g/mol)	log(K_oa_) (−)	k_p_g_ (m/h)	f_1_ (−)	TDI (µg/d/kg-bw)	Metabolite	MW_2_ (g/mol)	F_ue_ (−)
DEP	222	8.21	3.4	0.0414	500	MEP	194	0.69^a^
DnBP	278	9.83	4.8	0.0314	10	MnBP	222	0.69
DiBP	278	9.62	4.8	0.0243	10^c^	MiBP	222	0.69^a^
BBzP	312	11.6	5.9	0.0143	500^b^	MBzP	256	0.73
DEHP	391	12.9	5.8	0.0021	50^b^	MEHP	294	0.059
						MEHHP	294	0.233
						MEOHP	292	0.15
						MECPP	308	0.185

See text for description of variables.

a No excretion factor available at the time of data analysis; factor for MnBP was used [67].

b For comparison: Reference dose value RfD for BBzP is 200 µg/d/kg-bw, for DEHP 20 µg/d/kg-bw [97],[98].

c putative TDI by analogy to DnBP.

The potential pathways of exposure are ingestion, inhalation and dermal absorption. Dietary ingestion has long been believed to constitute the major source of exposure to high molecular weight phthalates. Ingestion of dust and, to some extent, personal care products, as well as mouthing of toys and other articles, may further contribute to ingestion exposures. Inhalation of air, airborne particles and aerosols from various sprays may also make a meaningful contribution. Dermal exposure through the use of personal care products and dermal contact with plastic products, soil and dust can add to the total intake of certain phthalates [41],[42],[43],[44],[45],[46],[47],[48],[49]. Additionally, recent studies argue that air-to-skin transdermal uptake may be a meaningful pathway for lower molecular weight phthalates [50],[51],[52],[53],[54]. This phthalate exposure pathway has only been included in one earlier study [54]. The relative contribution of each pathway to an individual’s total exposure varies with the phthalate and the age of the exposed individual [41],[43]. Infants and children tend to be more exposed than adults. This may partially be a consequence of added skin contact with surfaces and frequent mouthing of fingers and other objects such as plastic toys. The higher rate of dust ingestion and ingestion of phthalates present in breast milk, infant formula, cow’s milk or food packaging may further lead to higher phthalate exposures for children [7],[55]. In indoor settings, phthalates partition between the gas-phase and airborne particles, settled dust and exposed surfaces, including exposed skin and hair [56],[51]. The contribution to total exposure from exposures that occur in the indoor environment can be substantial for certain phthalates and age groups [41],[43].

Current limit values are mainly based on oral intake of phthalates. Limit values for intake via other pathways do not exist. However, absorption, distribution, and elimination of a chemical in the body differ for different exposure pathways [57],[58],[59]. Ingested compounds pass through the intestines and liver before entering the blood. Inhaled contaminants first pass through the lungs. Chemicals penetrating the skin can directly enter the blood. For nominally comparable exposures, the resulting biologically effective dose to various organs can be different for different exposure routes [60]. Therefore, understanding the contribution of each pathway to the total intake may well be important from a health-effects perspective.

Improved understanding of the metabolism and elimination of phthalates makes it possible to estimate total daily phthalate intakes, from all pathways and sources, based on the concentrations of phthalate metabolites in the urine [61],[62],[63],[64],[65],[66]. During the last decade there has been a substantial increase in studies that have used this approach to investigate phthalate exposures [67],[68]. Studies using human biomonitoring often only attempt to determine the total intake or intake from a specific source such as diet or personal care products [69],[70],[45]. Several studies have attempted to estimate phthalate intakes from different sources and pathways using various models [71],[72],[73],[74],[75],[76],[41],[42],[77],[52],[53]. The study closest in design to the present study is that by Guo and Kannan [43], which estimated phthalate intakes from dust ingestion, inhalation and dermal uptake (only considering dust adhered to skin) after measuring phthalate levels in dust collected from homes. The results were then compared to total intakes estimated from metabolites measured in urine. However, the study populations for urine sampling and dust sampling were not identical. Nor did the authors have information on physical characteristics, such as body weight. Thus, the analyses were performed using median values of the measured parameters coupled with recommended exposure factors. All indoor exposures were assumed to occur in the same environment. We are unaware of any study that has measured phthalate levels in dust and metabolite levels in urine for the same study population, and compared phthalate intakes from co-occurring exposure pathways to total intakes on a person-by-person basis.

The Danish *Indoor Environment and Children’s Health (IECH)* study is an investigation of potential associations between different indoor environmental factors and children’s health, especially allergies and asthma [78]. As part of this study, detailed investigations of the living environments of 200 children with parental-reported asthma and/or allergy (cases) and 300 randomly selected bases between 3 and 6 years of age were performed. These included collection of settled dust samples from both their bedrooms and daycare centers (DCC). The children also received a detailed examination by a medical doctor, at which time urine samples were collected from 441 of the children. Langer et al. [79] reports the mass fractions of five phthalate esters in the dust samples – DEP, DnBP, DiBP, BBzP and DEHP – and compares the results from the dust samples with those from other studies. Langer et al. [80] reports the concentration of eight metabolites of these phthalates in the urine – monoethyl phthalate (MEP) from DEP, mono-n-butyl phthalate (MnBP) from DnBP and BBzP, mono-isobutyl phthalate (MiBP) from DiBP, monobenzyl phthalate (MBzP) from BBzP, mono(2-ethylhexyl) phthalate (MEHP) from DEHP; and three secondary metabolites: mono-2-ethyl-5-hydroxyhexyl phthalate (MEHHP), mono-2-ethyl-5-oxohexyl phthalate (MEOHP) and mono-2-ethyl-5-carboxypentyl phthalate (MECPP), each from DEHP – and compares the results from the urine samples with those of other studies. The Langer et al. [80] paper also presents correlations between phthalate levels in dust collected from the children’s home and daycare center and the concentrations of metabolites in corresponding urine samples. The present paper builds on these data, critically examining the pathways by which children are exposed to phthalates in the indoor environment. We calculate total daily intakes (DI) of the target phthalates based on metabolite levels in the urine samples. For each child and each phthalate, we then compare these with estimated intakes from dust ingestion, inhalation and dermal absorption that occurred in the child’s home and daycare center. We conclude by comparing these intakes, estimated from either the urine samples or the indoor dust samples, with established Tolerable Daily Intake (TDI) values.

## Materials and Methods

### Ethics Statement

The study was approved by The Regional Scientific Ethical Committee for Southern Denmark (Case # S-20070108). Both verbal and written informed consent was obtained from the parents/guardians on behalf of all children participating in the study.

### Data Collection and Estimated Intakes

Between March and May 2008, dust samples were collected from the homes of 500 children and from the 151 daycare centers that these children attended in Odense, Denmark. Morning urine samples from 441 children were collected between August 2008 and April 2009. Detailed descriptions of the study population, dust sampling, urine sampling and their chemical analyses are presented elsewhere [78],[79],[80]. These studies also report the mass fractions of phthalates in the dust and the levels of phthalate metabolites in the urine (see Table S1 for a summary of these results).

In the present paper, the daily intake of the target phthalates, normalized for body weight, has been calculated for each child from the phthalate metabolite concentration in the urine (*DI_urine_*). Using the mass fractions of phthalates in the dust collected from the children’s homes and daycare centers, the co-occurring airborne concentrations (both gas phase and associated with airborne particles) of these phthalates were estimated. Next the children’s intakes of the target phthalates via inhalation, dust ingestion and dermal absorption were estimated for both the home and daycare environments. The total daily intake from the indoor environment was then calculated for the day of week the urine sampling occurred (*DI_indoors_*). Additionally, the total weekly intake (WI) from the indoor environment was calculated (*WI_indoors_*), and one-seventh of this value was termed the average daily intake from a week-long exposure. These analyses were conducted for 431 children from whom urine samples were collected and for whom dust samples from both home and daycare center were available.

### Daily Intakes from Urinary Phthalate Metabolite Concentrations (DI_urine_)

The daily intake of phthalates was estimated for each child from the concentrations of metabolites in its morning urine sample using equation (1) - the urinary volume-based calculation approach. We did not adjust our values using the creatinine correction approach for reasons outlined in Langer et al. [80].
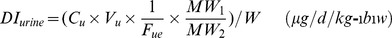
(1)where *DI_urine_* is the total daily intake normalized for body weight (unit: µg per day per kg of body weight; µg/d/kg-bw), *C_u_* is the urinary phthalate metabolite concentration (µg/L), *V_u_* is the daily excreted urinary volume (L/d) calculated from the estimated daily urine excretion rate of 22.4 mL/kg body weight [81],[82] and the child’s body weight (*W; kg*), *F_ue_* is the urinary excretion factor which describes the molar ratio between the excreted amount of a metabolite in relation to the intake of the parent phthalate (values taken from Table 1 of Wittassek et al. [68]), *MW_1_* and *MW_2_* are the molar weights of the parent phthalate and its metabolite, respectively (g/mol). The body weight of the children was measured on the day of urine sampling.

The final total daily intake of DEHP was calculated as the average intake obtained from equation (1) for the secondary metabolites of DEHP (MEHHP, MEOHP and MECPP). MEHP was not used to estimate the daily intake of DEHP since it is prone to contamination, has a much shorter elimination half-life and tends to be present at lower concentrations than the secondary metabolites [83],[84],[85],[86],[68],[87],[88],[89].

### Day-specific Intakes from Indoor Environment (DI_indoors_)

The intakes from dust ingestion, inhalation and dermal exposure were first determined separately for the home and daycare environments, using the equations in Table 2. In making these calculations, we assumed that a child spent 20 hours a day in their home and daycare environments. Four hours were assumed to be spent in transit, outdoors and in other indoor environments. We further assumed that: a child spent 9 hours asleep; on a weekday a child spent 8 hours in daycare, 6 of which were indoors; on a weekday a child spends 14 hours at home, of which the child was awake 5 hours; on a weekend all 20 hours indoors were spent at home, of which the child was awake 11 hours.

**Table 2:** Equations used in the calculation of daily intakes (µg/d/kg-bw) of phthalates by dust ingestion, inhalation and dermal exposure.

Pathway	No.	Equation	Parameters
Dust ingestion	(2)	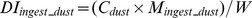	*C_dust_* is the mass fraction of phthalates in dust (µg/g), *M_ingest_dust_* is the daily dust ingestion rate (g/day)
Inhalation	(3)	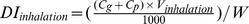	*(C_g_+C_p_)* is the total airborne concentration of a phthalate - both the gas phase concentration (C_g_) and the mass sorbed to particles (C_p_) per unit volume of air (ng/m^3^), *V_inhalation_* is the daily volume of air inhaled (m^3^/d)
Dermal exposurethrough air	(4)	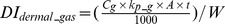	*k_p_g_* is the indoor air transdermal permeability coefficient (m/h), *A* is the child’s body surface area (m^2^), *t* is the daily duration of exposure (h)
Dermal exposurethrough dustadhered to skin	(5)	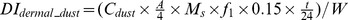	*A/4* is the skin surface area contaminated with dust particles, *M_s_* is the amount of dust adhered to skin (g/m^2^), *f_1_* is the fraction of phthalates transferred through the skin into the body, 0.15 is the fraction of phthalates in dust adhered to skin available for absorption (matrix effect)
Other	(6)	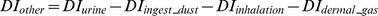	

See text for further details on the parameters used in the equations.

The mass fractions of phthalates in the dust collected from the home or daycare center (*C_dust_*) have been published elsewhere [79]. The daily dust ingestion rate (*M_ingest_dust_*) was assumed to be 60 mg/day [90]. Assuming that a child does not ingest dust while sleeping, the 60 mg of dust ingested in a day occurs during the 15 waking hours (4 mg/hour). This prorates to 44 mg/day ingested during the 11 hours spent awake in the home and daycare environments. We assumed that during weekdays a child ingests 20 mg/day (4 mg/hour × 5 hours) of dust at home and 24 mg/day (4 mg/hour × 6 hours) in daycare, while during the weekend 44 mg/day is ingested in the home.

The total daily inhalation rate (*V_inhalation_*) of a child between 3 and 6 years of age was assumed to be 10.9 m^3^/d [90]. Based on short-term inhalation rates stratified by activity level in the EPA’s child-specific exposure factors handbook, we estimated that the volume of air inhaled during the 6 hours spent indoors in daycare was 2.8 m^3^ (3 hours sedentary and 3 hours light activity), while the volume inhaled during the 14 hours spent indoors at home on a weekday was 5.2 m^3^. During a Saturday or Sunday, the volume inhaled during the 20 hours spent indoors at home was estimated to be 8 m^3^.

DEP is the most volatile of the five targeted phthalates. In the air it exists almost entirely in the gas phase and its presence in airborne particles makes a negligible contribution to its overall airborne concentration. In the present study, the gas phase concentration of DEP, *C_g_* (DEP), was estimated by assuming a linear relationship between its mass fraction in settled dust, *C_dust_*(DEP), and its gas phase concentration [56]. We used data from three previous studies that had made simultaneous measurements of DEP’s mass fraction in settled dust and its airborne concentration [75],[91],[92] to establish the parameters that defined the approximately linear relationship:
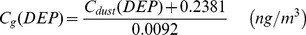
(7)where the units for *C_dust_*(DEP) are µg/g. The coefficient of determination for the relationship was R^2^ = 0.99.

The four other targeted phthalates, DnBP, DiBP, BBzP and DEHP, have meaningful concentrations in both the gas phase and in airborne particles, *C_p_*. In the case of these phthalates, we began by assuming that the logarithm of *(C_dust_/C_g_)* for a given phthalate was a linear function of the logarithm of its octanol-air partition coefficient, log(*K_oa_*) [56]. We used data from four previous studies [75],[91],[92],[93] to establish the parameters that defined this approximately linear relationship:
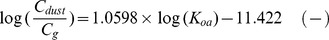
(8)


The coefficient of determination for the resulting relationship was R^2^ = 0.94. Using this approach, we could calculate *C_dust_/C_g_* for each phthalate, and then estimate its gas phase concentration in either a home or daycare center from its mass fraction measured in the dust sample taken from the home or daycare center.

For these four phthalates, we also needed to estimate their *total* airborne concentrations (*C_g_+C_p_*). To do this we assumed that the gas phase concentration of a given phthalate was related to its particle phase concentration through a partition coefficient, *K_p_*, and that the relationship between its gas phase concentration and its total airborne concentration was given by equation (9) [56]:
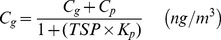
(9)where *TSP* is the average indoor mass concentration of airborne particles, assumed to be 20 µg/m^3^. *K_p_* for a given phthalate was estimated using equation (10) [56]:
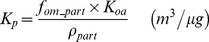
(10)where fom_part is the volume fraction of organic matter associated with airborne particles, assumed to be 0.4 (-) [94]; Koa is the phthalate’s octanol-air partition coefficient (-) [56]; and ρpart is the density of airborne particles, assumed to be 1 × 10−12 µg/m3.

The daily intake of phthalates via dermal absorption was estimated for two pathways: i) transport of gas-phase phthalates through the air to the skin and then through the epidermis into dermal capillaries (*DI_dermal_gas_*, eq. (4) in Table 2) and ii) transport from dust adhered to the skin into dermal capillaries (*DI_dermal_dust_*, eq. (5) in Table 2). The former was estimated using an approach outlined in Weschler and Nazaroff [51], using the indoor air transdermal permeability coefficient (*k_p_g_*), which describes the transport of a gas-phase phthalate from air in the core of a room through the boundary layer adjacent to skin and then through the stratum corneum/epidermis to dermal capillaries. The child’s body surface area was calculated from the child’s weight and height using the relationship reported by Dubois and Dubois [95]:
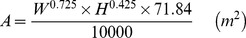
(11)where *H* is the child’s height (cm).

When calculating *DI_dermal_dust_*, we assumed only 25% of the total skin surface area to be contaminated with dust particles. The amount of dust adhered to the skin (*M_s_*) was assumed to be 0.96 g/m^2^ [43]. To account for reduced absorption caused by physical-chemical bonding to dust (the matrix effect), we used a coefficient of 0.15, which represents the fraction of phthalates available for dermal absorption [96]. The fraction of the available phthalate actually transferred through the skin into the body (*f_1_*) [41] is shown for each phthalate in Table 1, which lists selected physical-chemical properties of the five target phthalates as well as their tolerable daily intakes (TDI). Also listed are selected properties of their metabolites. Preliminary analysis indicated that the contribution to total intake resulting from phthalates associated with dust adhered to skin was negligible; hence *DI_dermal_dust_* was not included in further analyses (see Results and Discussion).

Phthalate metabolism occurs on a scale of hours [99],[100],[89],[101]. All urine samples were collected in the morning after the child awoke. In the case of a urine sample collected on a Monday (n = 120) or Sunday (n = 1), metabolites resulting from daycare exposures occurring on Friday had presumably been excreted prior to urine collection, and the final day-specific phthalate intake (*DI_pathway_final_*) was based solely on exposures occurring in the home during the weekend. For children whose urine samples were taken on other days, the final intake resulting from indoor exposures was calculated by combining the intakes that occurred in the daycare and home environments. For the purpose of comparing intakes estimated for indoor pathways with total daily intakes estimated from urine samples (*DI_urine_*), we roughly corrected for metabolism and excretion that occurred during the 24 hours prior to urine sampling. As the exposure in the daycare center occurred ∼15 hours before urine sampling (about two or more half-lives for most of the metabolites) we reduced the contribution from the 6-hour indoor exposure in the daycare by a factor of four:
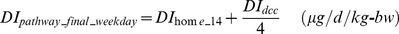
(12)where *DI_home_14_* is the daily intake from 14 hours of day- and nighttime exposure in the home (occurring both on weekdays and weekends) and *DI_dcc_* is the daily intake from 6 hours of exposure in the daycare center during the day before urine sampling. In order to produce comparable results for the weekends, we similarly reduced an identical portion of the intake from the home during the weekend:
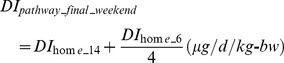
(13)where DIhome_6 is the daily intake from 6 hours of daytime exposure in the home.

For a given child, daily phthalate intake resulting from indoor exposures on the day prior to urine sampling was calculated as the sum of intakes by three exposure pathways:
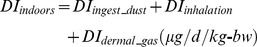
(14)


The net intake from other pathways such as diet, the outdoor environment or indoor environments other than the home and daycare (*DI_other_*) was calculated from equation (6) in Table 2.

### Average Daily Intake from a Week-long Exposure (WI_indoors/_7)

To estimate the average daily phthalate intake from the indoor environment, regardless of the time of urine sampling, we first calculated the weekly intake using the following weighting scheme for each pathway:
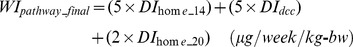
(15)where *DI_home_14_* is the daily intake from 14 hours of day- and nighttime exposure in the home during weekdays, *DI_dcc_* is the daily intake from 6 hours in the daycare during weekdays and *DI_home_20_* is the daily intake from 20 hours spent in the home during Saturday or Sunday (weekend). The weekly intakes for each pathway were then summed into *WI_indoors_* similar to equation (14). One-seventh of this value is the average daily intake from a week-long exposure. Additionally, we determined the fraction of the total weekly intake (*WI_indoors_*) attributable to each pathway. We also calculated the fraction of *WI_indoors_* and the fraction of the weekly intake from each pathway (*WI_pathway_final_*) attributable to the home and daycare environments separately.

The results of *DI_urine_* and *WI_indoors/_7* were compared to the tolerable daily intake (TDI) values derived by the European Food Safety Authority for BBzP, DnBP and DEHP [102],[103],[104]. In the case of DiBP, since there is no established limit value, the TDI for DnBP was used. The TDI value for DEP was taken from a statement on dietary exposure to phthalates by the Committee on Toxicity of Chemicals in Food, Consumer Products and Environment [105]. Based on different health effects, the US EPA recommends a daily oral Reference Dose (RfD) lower than the TDI for BBzP and DEHP [97],[98]. We also compare our intake levels of these phthalates to the EPA’s RfDs.

To evaluate the cumulative exposure to several endocrine active (anti-androgenic) phthalates that may act in a dose-additive manner, we applied the concept of a relative cumulative Tolerable Daily Intake (*TDI_cum_*) introduced by Koch et al. [26]. A *TDI_cum_* above 100% indicates that the cumulative daily phthalate intake surpasses the tolerable levels. As suggested by Koch et al. [26], we restricted our analyses to phthalates whose TDIs were based on the same health endpoint (DnBP, DiBP and DEHP):
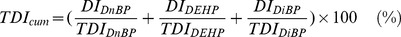
(16)


The *TDI_cum_* was determined both for phthalate intakes obtained from the urinary phthalate concentrations (*DI_urine_*) and for the average daily intakes from a week-long exposure (*WI_indoors/_*7). Spearman correlation coefficients were calculated between *DI_urine_* and *DI_indoors_.* Statistical analyses were done in STATA software, release 11.0 for Windows (StataCorp LP, College Station, Texas, USA).

## Results

**Figure 1:**
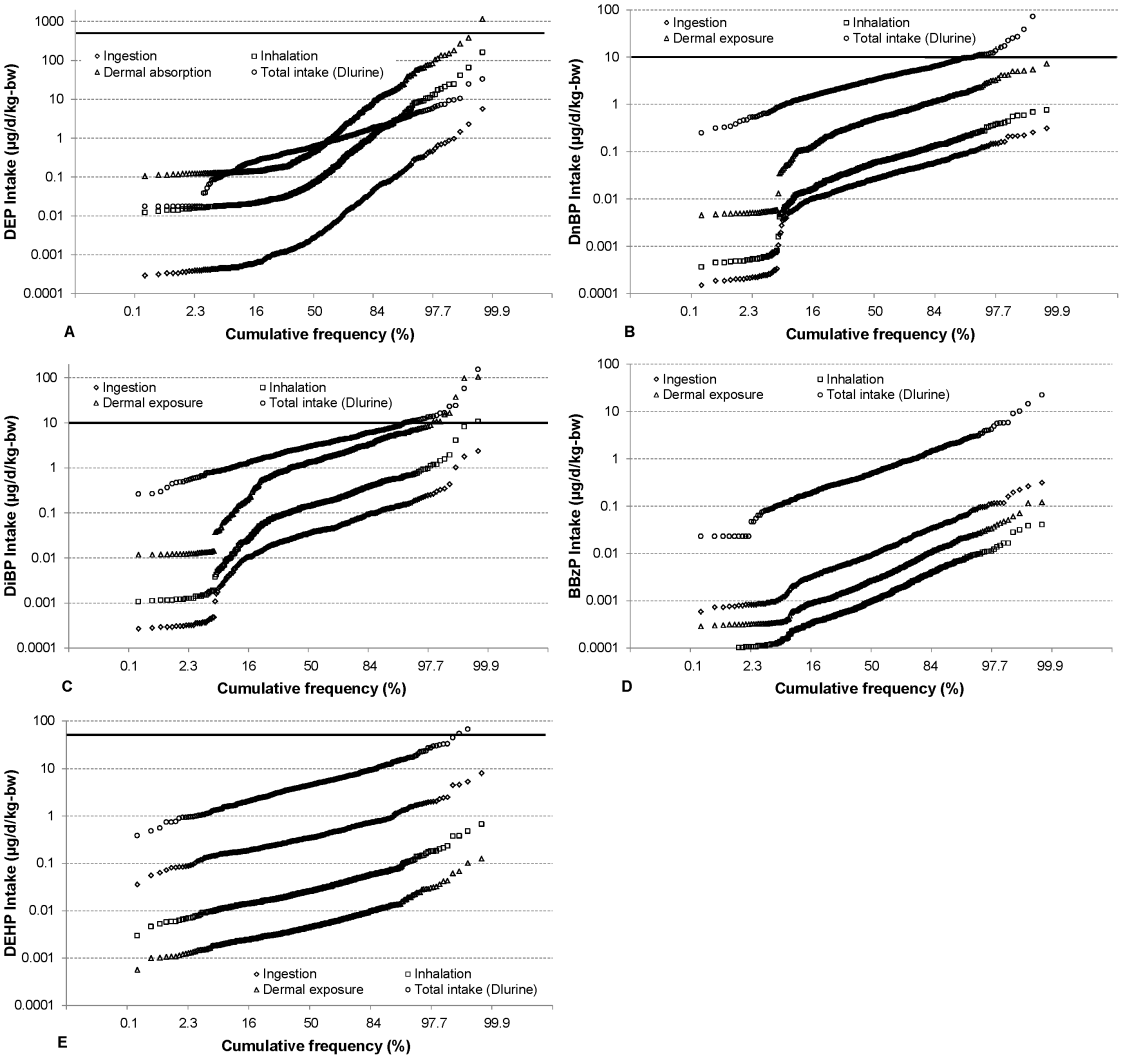
Cumulative frequency distributions for the estimated intakes of phthalates. Distributions are shown for the intakes on the day before urine sampling by different indoor exposure pathways and the total intakes calculated from the metabolite concentrations measured in urine (*DI_urine_*). A) DEP, B) DnBP, C) DiBP, D) BBzP and E) DEHP. The solid horizontal line indicates the TDI value. The TDI value for BBzP (500 µg/d/kg-bw) is not indicated in the plot.

Figures 1A through 1E present distribution plots of the total intakes (*DI_urine_*) and intakes on the day before urine sampling from the three pathways for the individual phthalates (logarithmic scale for the y-axis) against the cumulative distribution function with the x-axis scaled according to the normal error function. For a given phthalate, the distribution curves for all three pathways have roughly the same slope. Comparable distribution slopes were observed for intakes calculated from urine and those calculated from dust mass fractions. The only exceptions were the slopes of the daily intakes for DEP from the three indoor exposure pathways, which were larger than the slope of the intake calculated from urine. The slope of the distribution of dust mass fractions of DEP was also larger than that of other phthalates, presumably reflecting a wide variation in the use of personal care products [79]. For some of the phthalates, the day-specific intakes via ingestion, inhalation and dermal absorption had skewed tails, reflecting a number of dust mass-fraction values below the detection limit. In contrast, distributions without skewed tails were obtained for the average daily intakes estimated from a week-long exposure (*WI_indoors/_*7), which were determined based on exposure in both environments for all children, regardless of the day of urine sampling (Figure S1). Apart from the tails, most of the plots are approximately linear, indicating that the distributions are better described as “log-normal” rather than “normal”. Therefore, in what follows we refer to the median values when describing the results.

Table 3 compares total daily intakes estimated from metabolite concentrations in the urine samples (*DI_urine_*) with those estimated from dust ingestion, inhalation and dermal absorption occurring on the day before urine sampling. Descriptive statistics for the sum of the three indoor pathways are also presented in this table. Total daily intake, as estimated from the urine samples, was the highest for DEHP. Comparing medians, daily intakes for DnBP and DiBP were around 70% of that for DEHP, while DEP and BBzP intakes were considerably lower. The daily total intake from indoor exposures (dust ingestion, inhalation and dermal absorption) was the lowest for BBzP and the highest for DiBP. The largest intake resulting from dust ingestion was that for DEHP, while the largest by inhalation and dermal absorption was the intake of DiBP. Statistically significant positive Spearman correlation coefficients were obtained between the total daily intakes (*DI_urine_*) and the corresponding day-specific daily intakes from the three pathways (*DI_indoors_*) for DEP, DnBP, DiBP and BBzP (Table 4). Table 5 summarizes the descriptive statistics for the average daily intake as estimated from a week-long exposure via each “indoor” pathway. Although these intakes via all pathways, as well as the corresponding total indoor intakes, were higher than their day-specific counterparts, the trends described above are similar in Tables 3 and 5.

**Table 3:** Descriptive statistics regarding the intake of five phthalates (µg/d/kg-bw) calculated from urinary metabolite concentrations and estimated from exposure via three pathways in the indoor environment on the day before urine sampling.

		DEP	DnBP	DiBP	BBzP	DEHP
*DI_urine_*	Mean (SD)	1.19 (2.33)	4.65 (9.07)	4.13 (8.19)	0.91 (1.63)	7.4 (26.2)
	GM (GSD)	0.61 (3.2)	3.18 (2.2)	2.83 (2.2)	0.50 (2.9)	4.49 (2.3)
	Min	0.017	0.25	0.26	0.023	0.38
	Max	33.0	162.9	152.4	22.3	533.3
	Median	0.62	3.26	2.93	0.49	4.42
	95^th^ %tile	3.89	10.03	10.02	2.79	16.9
*DI_ingest_dust_*	Mean (SD)	0.057 (0.32)	0.04 (0.04)	0.062 (0.16)	0.02 (0.03)	0.51 (0.65)
	GM (GSD)	0.005 (7.3)	0.02 (3.9)	0.027 (4.4)	0.01 (3.4)	0.37 (2.1)
	Min	0.0003	0.0002	0.0003	0.0006	0.04
	Max	5.63	0.31	2.36	0.32	8.09
	Median	0.003	0.03	0.035	0.009	0.35
	95^th^ %tile	0.20	0.10	0.16	0.075	1.53
*DI_inhalation_*	Mean (SD)	1.56 (8.91)	0.081 (0.094)	0.26 (0.70)	0.002 (0.004)	0.04 (0.05)
	GM (GSD)	0.13 (6.8)	0.043 (4.0)	0.1 (4.8)	0.001 (3.4)	0.028 (2.1)
	Min	0.012	0.0004	0.001	0.000	0.003
	Max	159.4	0.76	10.7	0.04	0.67
	Median	0.071	0.058	0.14	0.001	0.026
	95^th^ %tile	5.76	0.24	0.66	0.009	0.11
*DI_dermal_gas_* a	Mean (SD)	10.9 (63.7)	0.73 (0.85)	2.44 (7.26)	0.006 (0.011)	0.007 (0.01)
	GM (GSD)	0.89 (7.0)	0.39 (4.1)	0.95 (4.9)	0.003 (3.4)	0.005 (2.1)
	Min	0.11	0.005	0.012	0.0003	0.0006
	Max	1175.8	7.26	104.5	0.12	0.13
	Median	0.45	0.51	1.33	0.003	0.005
	95^th^ %tile	41.1	2.13	6.15	0.024	0.02
*DI_indoors_* b	Mean (SD)	12.5 (72.9)	0.84 (0.98)	2.75 (8.11)	0.029 (0.047)	0.56 (0.71)
	GM (GSD)	1.03 (6.9)	0.45 (4.0)	1.08 (4.9)	0.01 (3.4)	0.41 (2.1)
	Min	0.12	0.005	0.013	0.001	0.04
	Max	1340.8	8.34	117.5	0.47	8.88
	Median	0.53	0.60	1.52	0.01	0.37
	95^th^ %tile	47.1	2.49	6.99	0.10	1.63
*DI_other_* c	Mean (SD)	−11.3 (72.6)	3.81 (9.10)	1.38 (11.4)	0.88 (1.62)	6.84 (26.2)
	GM (GSD)	0.40 (3.7)^d^	2.43 (2.9)^d^	1.81 (3.4)^d^	0.47 (3.1)^d^	3.80 (2.7)^d^
	Min	−1334.0	−3.49	−113.0	−0.06	−3.94
	Max	32.7	162.7	146.2	22.2	532.9
	Median	−0.022	2.59	1.24	0.46	3.94
	95^th^ %tile	1.81	9.56	8.09	2.74	16.6

SD-standard deviation, GM-geometric mean, GSD-geometric standard deviation.

a Due to negligible contribution, detailed data for *DI_dermal_dust_* are not presented. Its medians are: 0.00007 (DEP), 0.0005 (DnBP), 0.0006 (DiBP), 0.00007 (BBzP), 0.0004 (DEHP).

b Sum of *DI_ingest_dust_, DI_inhalation_, DI_dermal_gas_* on a child-by-child basis.

c *DI_other_ = DI_urine_ – DI_ingest_dust_ – DI_inhalation_ – DI_dermal_gas._*

d Based on positive values only: n = 214 (DEP), 403 (DnBP), 313 (DiBP), 429 (BBzP), 423 (DEHP).

**Table 4:** Spearman correlation coefficients between phthalate intakes calculated from urinary phthalate metabolite concentrations (*DI_urine_*) and from the exposure to phthalates via three pathways in the indoor environment on the day before urine sampling (*DI_indoors_*).

		*DI_urine_*				
		DEP	DnBP	DiBP	BBzP	DEHP
*DI_indoors_*	DEP	0.30^a^	0.073	0.047	0.021	0.041
	DnBP	0.092	0.14^b^	0.007	0.076	0.113^c^
	DiBP	0.103^c^	0.052	0.12^c^	0.02	0.009
	BBzP	0.068	0.098^c^	0.011	0.21^a^	0.044
	DEHP	0.02	0.055	−0.071	0.068	0.026

a p<0.001,

b p<0.005,

c p<0.05.

**Table 5:** Descriptive statistics of the average daily phthalate intake (µg/d/kg-bw) from a week-long exposure via three pathways in the indoor environment.

		DEP	DnBP	DiBP	BBzP	DEHP
*WI_ingest_dust/_7*	Mean (SD)	0.074 (0.42)	0.076 (0.067)	0.092 (0.21)	0.041 (0.054)	1.04 (0.94)
	GM (GSD)	0.009 (5.6)	0.056 (2.3)	0.055 (2.6)	0.024 (2.7)	0.83 (1.8)
	Min	0.0006	0.0004	0.0005	0.001	0.15
	Max	7.57	0.50	3.19	0.36	8.86
	Median	0.005	0.055	0.057	0.022	0.78
	95^th^ %tile	0.27	0.22	0.21	0.12	2.85
*WI_inhalation/_7*	Mean (SD)	1.78 (10.2)	0.13 (0.12)	0.32 (0.80)	0.004 (0.005)	0.061 (0.063)
	GM (GSD)	0.19 (5.7)	0.093 (2.4)	0.17 (3.0)	0.002 (2.7)	0.048 (1.9)
	Min	0.019	0.0008	0.002	0.0001	0.009
	Max	183.9	0.94	12.4	0.045	0.70
	Median	0.11	0.094	0.19	0.002	0.043
	95^th^ %tile	6.66	0.35	0.80	0.012	0.17
*WI_dermal_gas/_7*	Mean (SD)	12.1 (71.2)	1.10 (1.04)	2.91 (8.13)	0.009 (0.013)	0.010 (0.011)
	GM (GSD)	1.25 (5.9)	0.77 (2.4)	1.49 (3.1)	0.005 (2.8)	0.008 (1.9)
	Min	0.15	0.007	0.016	0.0004	0.002
	Max	1319.9	8.71	117.6	0.14	0.13
	Median	0.69	0.81	1.69	0.005	0.007
	95^th^ %tile	46.2	3.04	7.09	0.034	0.029
*WI_indoors_* ^/^ *7* a	Mean (SD)	13.9 (81.9)	1.30 (1.22)	3.32 (9.13)	0.054 (0.071)	1.11 (1.01)
	GM (GSD)	1.45 (5.9)	0.92 (2.4)	1.72 (3.0)	0.032 (2.7)	0.89 (1.8)
	Min	0.17	0.008	0.018	0.002	0.16
	Max	1511.4	10.1	133.2	0.54	9.69
	Median	0.80	0.97	1.95	0.030	0.83
	95^th^ %tile	53.1	3.50	7.97	0.16	3.07

SD-standard deviation, GM-geometric mean, GSD-geometric standard deviation.

a *WI_indoors_* is the sum of *WI_ingest_dust_, WI_inhalation_, WI_dermal_gas_* on a child-by-child basis.

The three indoor exposure pathways accounted for a meaningful fraction of the total daily intakes (*DI_urine_*) of DEP, DnBP and DiBP (Table 6). The entire DEP intake could be explained by exposures in the indoor environment, while indoor exposures were responsible for ∼50% and ∼17% of the DiBP and DnBP intake, respectively. For all three phthalates, dermal absorption was by far the major route of intake indoors, while inhalation was roughly 1/10^th^ of the dermal pathway and dust ingestion an even smaller percentage. For BBzP and DEHP, the indoor exposure pathways made smaller contributions to the total intake than was the case for DEP, DnBP and DiBP. For these higher molecular weight phthalates, dust ingestion made the largest contribution of the indoor pathways. Although not directly comparable, the total daily indoor intakes estimated from a week-long exposure (*WI_indoors/_*7) would explain 159% of *DI_urine_* for DEP, 28% for DnBP, 65% for DiBP, 6% for BBzP and 19% for DEHP (data not shown), compared to 102%, 17%, 50%, 3% and 8% explained by *DI_indoors_*, respectively.

**Table 6:** Contribution (%) of each exposure pathway on the day before urine sampling to the daily intake estimated from urinary metabolite concentrations (*DI_urine_*).

		DEP	DnBP	DiBP	BBzP	DEHP
*DI_ingest_dust_/DI_urine_ ×100*	Mean (SD)	7.0 (26.1)	1.5 (2.7)	2.7 (7.2)	5.1 (9.8)	15.0 (21.3)
	GM (GSD)	0.8 (7.5)	0.7 (4.6)	1.0 (5.1)	2.0 (4.2)	8.3 (3.0)
	Min	0.004	0.002	0.002	0.02	0.06
	Max	379	39.2	111	128	175
	Median	0.50	0.8	1.2	2.0	7.6
	95^th^ %tile	29.3	5.0	8.8	19.1	52.3
*DI_inhalation_/DI_urine_ ×100*	Mean (SD)	189 (714)	3.2 (5.9)	11.0 (31.9)	0.6 (1.2)	1.1 (1.8)
	GM (GSD)	21.5 (7.1)	1.4 (4.6)	3.6 (5.5)	0.2 (4.2)	0.6 (3.0)
	Min	0.1	0.005	0.009	0.003	0.005
	Max	10665	87.6	501	16.3	14.6
	Median	12.8	1.6	4.6	0.2	0.6
	95^th^ %tile	830	11.4	37.2	2.3	3.9
*DI_dermal_gas_/DI_urine_ ×100*	Mean (SD)	1300 (4772)	28.9 (54.1)	105 (341)	1.5 (3.1)	0.2 (0.3)
	GM (GSD)	147 (7.2)	12.1 (4.6)	33.5 (5.6)	0.6 (4.2)	0.1 (3.0)
	Min	0.80	0.06	0.1	0.01	0.001
	Max	69469	806	6036	40.8	3.0
	Median	89.2	14.2	44.0	0.6	0.1
	95^th^ %tile	5279	108	338	6.5	0.7
*DI_indoors_* a */DI_urine_ ×100*	Mean (SD)	1497 (5510)	33.6 (62.5)	118 (379)	7.2 (14.0)	16.3 (23.4)
	GM (GSD)	169 (7.1)	14.2 (4.6)	38.1 (5.5)	2.8 (4.2)	9.0 (3.0)
	Min	0.9	0.07	0.1	0.03	0.07
	Max	80513	933	6648	185	192
	**Median**	**102**	**16.5**	**49.9**	**2.9**	**8.2**
	95^th^ %tile	6021	122	382	27.6	57.0

SD-standard deviation, GM-geometric mean, GSD-geometric standard deviation.

a Sum of *DI_ingest_dust_, DI_inhalation_, DI_dermal_gas_* on a child-by-child basis.

More than 80% of the weekly intake of DEP, DnBP and DiBP from the indoor environment was attributable to dermal absorption of these compounds from the air (Table 7). Another ∼10% came from inhalation, while dust ingestion contributed very little to the total intake. On the other hand, 75% and 95% of the weekly indoor intake of BBzP and DEHP, respectively, entered the body via dust ingestion. The exposure to gas-phase BBzP through skin was responsible for ∼17% of the total intake from the three pathways. For all phthalates, the intake through dermal contact with adhered dust (*WI_dermal_dust_*) contributed negligibly to the total intake (<1%).

**Table 7:** Contribution (%) of each exposure pathway to the weekly intake from indoors (*WI_indoors_*).

		DEP	DnBP	DiBP	BBzP	DEHP
*WI_ingest_dust_/* *WI_indoors_* a *×100*	GM	0.6	6.0	3.2	75.6	93.5
	Median	0.6	6.0	3.0	76.8	93.6
*WI_inhalation_/* *WI_indoors_* a *×100*	GM	13.2	10.0	9.8	6.5	5.4
	Median	13.2	10.0	9.7	6.4	5.4
*WI_dermal_gas_/* *WI_indoors_* a *×100*	GM	86.0	83.6	86.8	16.7	0.9
	Median	86.1	83.9	87.1	16.5	0.9
*WI_dermal_dust/_* *WI_indoors_* a *×100*	GM	0.01	0.08	0.04	0.5	0.09
	Median	0.01	0.08	0.04	0.4	0.09

GM-geometric mean.

a To indicate the negligible contribution of WI_dermal_dust_, WI_indoors_ here is the sum of WI_ingest_dust_, WI_inhalation_, WI_dermal_gas_ and WI_dermal_dust._

More than 75% of *WI_indoors_* of DEP and DiBP came from exposure in the home (Table S2) as opposed to exposures in the daycare center. This is due to the relationship between the dust mass fractions of these phthalates and the exposure time in the two environments. For both phthalates, there was a large contribution from dermal absorption, which during the course of a week occurred for a longer time in the home. At the same time, the mass fraction of DEP in the dust was nearly the same in the homes and daycares (1.7 vs. 2.2 µg/g), while for DiBP the median mass fraction was slightly higher in the homes than in daycares (27 vs. 23 µg/g; [79]). With 84% of the DnBP intake coming from dermal exposure, a slightly higher fraction of the total DnBP intake originated in the home environment, even though the median dust mass fraction of DnBP was ∼2.5 times higher in the daycare centers than in the homes. The higher-molecular-weight phthalates mainly entered the body through dust ingestion, which, during weekdays, occurred to a larger extent in the daycare centers than in the homes (24 mg of ingested dust per day at daycare vs. 20 mg at home). The mass fractions of BBzP and DEHP in the dust were substantially higher in the daycares than in the homes. Consequently, 67% of the BBzP intake from indoors and 60% of the DEHP intake from indoors were attributable to the daycare environment.

**Figure 2:**
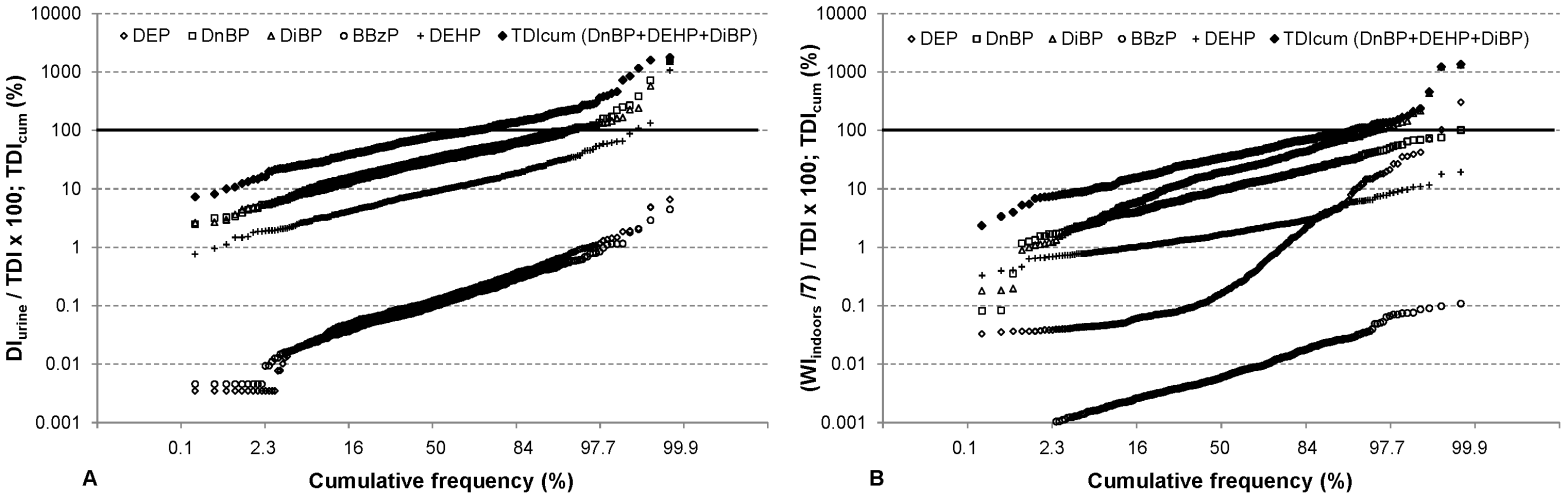
Cumulative frequency distribution of the ratio of the daily intake to the TDI for each of the five phthalates. The solid diamonds depict the relative cumulative tolerable daily intake (TDI_cum_) for DnBP, DiBP and DEHP; A) daily intakes calculated from the excreted amount of phthalates in the urine (*DI_urine_*), B) average daily intakes from a week-long exposure in the indoor environment through dust ingestion, inhalation and dermal absorption. Values above the solid horizontal line (100%) exceed the TDI of the given phthalate or the TDI_cum_ for the three phthalates.

The daily phthalate intakes occasionally exceeded the tolerable daily intake (TDI) established by the European Food Safety Authority (Figure 2; Table 8). In 23 children (5.3%) the daily intake of DiBP, determined from the urinary concentration of its monoester metabolite, exceeded the limit value; in 22 children the daily intake of DnBP exceeded its TDI; and in 3 children the daily intake of DEHP exceeded its TDI. None of the children had intakes that exceeded the TDI for DEP and BBzP. The median fractions of TDI reached by the total intake of phthalates were between 0.1% for DEP and BBzP and 33% for DnBP. Five percent of children with the highest intake of DnBP and DiBP had an intake above 100% and 102% of the TDI, respectively. The average daily intakes based on a week-long exposure in the indoor environment exceeded the TDI for fewer children. Interestingly, 14 children (3.2%) still exceeded the TDI for DiBP via the three indoor exposure pathways. These children were not identical to the ones that exceeded the TDI based on the urinary metabolite levels. The total DEHP intake of 16 children (3.7%) exceeded the RfD. The indoor environment contributed very little to the DEHP intake (see Table 6), and the children’s intake from the three indoor pathways did not exceed the RfD. None of the children had an intake of BBzP that exceeded its RfD.

**Table 8:** Portions (%) of the tolerable daily intakes (TDI), cumulative tolerable daily intake (TDI_cum_) and reference dose (RfD for DEHP) reached by the daily phthalate intake calculated from urinary concentrations (*DI_urine_*) and from a week-long exposure via three pathways in the indoor environment (*WI_indoors/_*7).

		DEP	DnBP	DiBP	BBzP	DEHP	TDI_cum_ a	*DEHP (RfD)*
*DI_urine/_TDI × 100*	#(%) above 100% b	0(0)	22(5)	23(5)	0(0)	3(0.7)	131(30)	*16(4)*
	Mean	0.2	47	41	0.2	15	103	*37*
	GM	0.1	32	28	0.1	9.0	75	*22*
	Min	0.003	2.5	2.6	0.005	0.8	7.3	*1.9*
	Max	6.6	1629	1524	4.5	1067	1773	*2667*
	Median	0.1	33	29	0.1	8.8	79	*22*
	95^th^ %tile	0.8	100	102	0.6	33	223	*84*
*(WI_indoors/_7)/TDI × 100*	#(%) above 100% b	2(0.5)	1(0.2)	14(3)	0(0)	0(0)	26(6)	*0(0)*
	Mean	2.7	13	33	0.01	2.2	48	*5.6*
	GM	0.3	9.2	17	0.006	1.8	33	*4.4*
	Min	0.03	0.08	0.2	0.0004	0.3	2.3	*0.8*
	Max	302	101	1330	0.1	19	1340	*48*
	Median	0.2	9.7	20	0.006	1.7	33	*4.2*
	95^th^ %tile	11	35	80	0.03	6.1	109	*15*

GM-geometric mean.

a Based on (DnBP+DiBP+DEHP).

b Number (percentage) of children whose values exceed the TDI, TDI_cum_ or RfD.

At the median level, the total intakes, as estimated from the urine samples, were 9% of the TDI for DEHP, 33% for DnBP and 29% for DiBP. These three phthalates had a median summed intake that was 79% of *TDI_cum_*. The 95^th^ percentile of the summed intake was 223% of *TDI_cum_*. 131 out of 431 children (30%) had a summed intake of these three phthalates that exceeded *TDI_cum_*, indicating that nearly every third child’s cumulative daily phthalate intake surpassed the tolerable levels (Figure 2; Table 8). Looking at the daily indoor intakes, based on one-week averages (*WI_indoors/_7*), the children’s summed median intake was approximately a third of *TDI_cum_*. A meaningful fraction of the children (26 out of 431 or 6%) had summed intakes from the three indoor pathways that exceeded *TDI_cum_*.

## Discussion

### Total Intake Based on Metabolites in Urine Samples

Although there are numerous studies of phthalate intakes for adults (see summary in Wittassek et al. [68] and recent results in Guo et al. [106], Ye et al. [107] and Chen et al. [108]), we will compare our phthalate intake estimates with those of studies that focused on children (Table 9). This approach is reasonable given the physiological and potential toxicokinetic differences, age-dependent metabolism and differences in exposure to phthalates between adults and children [41],[109],[110],[111],[112], [113]. Table 9 is based on estimates derived from metabolite excretions in urine, while Table S3 is based on estimates derived from exposure to different media (food, air, water, soil, dust; see part A of Discussion S1).

**Table 9:** Daily phthalate intakes (µg/d/kg-bw) of children calculated from urinary phthalate metabolite concentrations.

Reference/gender/age	Survey years	Country	n	DEP	DnBP	DiBP	BBzP	DEHP
Koch et al. and Wittassek et al. [63], [65],boys (2–14 yrs)	01–02	Germany	106	–	4.46^b^(7.04)	–	0.48^b^(0.91)	4.9^b^(8.4)
Koch et al. and Wittassek et al. [63], [65],girls (2–14 yrs)	01–02	Germany	133	–	4.74^b^(7.76)	–	0.31^b^(0.72)	3.9^b^(7.4)
Koch et al. [26], (5–6 yrs)	07	Germany	108	–	1.9^b^	2.1^b^	0.3^b^	4.5^b^
Lin et al. [114], (2–3 yrs)	03–04	Taiwan	30	–	3.81^a^	1.28^a^	0.17^a^	8.1^a^
Lin et al. [114], (5–6 yrs)	06–07	Taiwan	59	–	5.29^a^	0.91^a^	0.17^a^	10.9^a^
Guo et al. [106], boys (<10 yrs)	10	China	7	0.8	6.1	–	–	3.4
Guo et al. [106], girls (<10 yrs)	10	China	3	0.1	0.6	–	–	14.6
Frederiksen et al. [113], boys (6–10 yrs)	07	Denmark	25	0.92	5.27^j^		0.96	5.67
Frederiksen et al. [113], girls (6–10 yrs)	07	Denmark	24	1.13	5.28^j^		0.97	5.37
Frederiksen et al. [113] (6–21 yrs)	07	Denmark	129	1.09	4.29^j^		0.62	4.04
**This study, (3–6 yrs)**	**08–09**	**Denmark**	**431**	**0.62**	**3.26**	**2.93**	**0.49**	**4.42**
**Estimated daily phthalate intakes reported in Calafat and McKee** b [**115**]								
6–11 yrs^c^	99-00	USA	328	1.7^a^	–	–	–	0.6^a^
6–11 yrs^d^	99-02	USA	392	1.8^a^	–	–	–	0.6(2.4;2.6)h,a
3–14 yrs^e^	01-02	Germany	254	–	–	–	–	0.7(2.6;3.1)h,a
<7 yrs^f^	03	Germany	36	–	–	–	–	1.0(3.5;3.8)^h^
12–18mths^g^	00	USA	19	6.3^i^	–	–	–	2.8^i^

All values are medians except when indicated otherwise.

a Geometric Mean.

b Daily intakes from creatinine-based model, (from volume-based model in parentheses, when present).

c Estimated using urinary concentrations from Silva et al. [111].

d Estimated using urinary concentrations from CDC [116].

e Estimated using urinary concentrations from Becker et al. [112].

f Estimated using urinary concentrations from Koch et al. [110].

g Estimated using urinary concentrations from Brock et al. [117].

h from MEHP (MEHHP; MEOHP).

i Mean value.

j MnBP+MiBP.

Children’s phthalate intakes, calculated from urinary phthalate metabolite concentrations, are remarkably similar among the studies listed in Table 9. These studies were made in several countries and span the period from 2001 to 2010; the highest daily intakes were found for DEHP, and the lowest for BBzP. Using the volume-based method, slightly higher intakes of DnBP and DEHP were reported for Asian countries, as well as in Germany about a decade ago. The recent Danish study by Frederiksen et al. [113] provides the most straightforward comparison with our results. Although the children were somewhat older in the Frederiksen study, very similar intake values were obtained for most phthalates. The largest relative difference was for BBzP, where we observed intakes about half of those reported by the mentioned study. Currently we can only speculate about the reasons for this difference. One plausible explanation could be smaller exposure to BBzP due to differences in housing and lifestyle between residents of Odense and Copenhagen, where Frederiksen et al. [113] conducted their study. Dwellings in Copenhagen tend to be older. Due to lower turnover of materials in the Copenhagen buildings, they may contain more materials plasticized with BBzP. Moreover, while our study relied on first morning urine, the results in the Copenhagen study are based on 24-hour urine samples. Our results are also similar within a factor of two to those reported in a recent German study by Koch et al. [26]. However, these results were obtained with the creatinine-based method which can give lower results than the volume-based method, as was observed in an earlier German study [63],[65].

The median concentrations of the metabolites in the urine samples tended to be slightly higher in girls than in boys, except for the DEHP metabolites, which were slightly higher in boys. The differences were not statistically significant [80]. The total phthalate intakes (*DI_urine_*) had the same tendency (data not shown). Again, the differences were not significant (Student’s two sample t-test and Wilcoxon rank-sum test). These findings are in agreement with the results of Koch et al. [26] and Frederiksen et al. [113]. Koch et al. [63] and Wittassek et al. [65] found higher intake of DEHP and BBzP but not of DnBP for boys. We found no significant differences between boys and girls when comparing the daily intakes estimated from the phthalate mass fractions in the dust. Furthermore, we found only slight (and not statistically significant) differences between cases and bases when comparing daily intakes. Additional discussion regarding differences in daily intakes between cases and bases can be found in part B of Discussion S1.

Our intakes of DEP were lower while intakes of DEHP were somewhat higher than those estimated by Calafat and McKee [115] from urinary levels reported in other studies. The estimates of exposures to these phthalates by Calafat and Mckee [115] were made using geometric mean urinary phthalate metabolite concentrations and not on a child-by-child basis. The higher intakes of DEP in the US studies may reflect a different pattern of use for personal care products in the US compared to Denmark.

A reasonably good agreement was found between the fairly complete scenario-based exposure assessment by Wormuth et al. [41] and our total daily intakes with the largest difference obtained for DiBP (see Table S3). It is important to note, that according to CDC’s NHANES data, only the levels of MiBP in children’s urine have increased during the last decade, while metabolite concentrations of other phthalates have decreased during this time [109]. Similar trends have been reported by Wittassek et al. [66] and Göen et al. [118] based on data collected from university students in Germany over a period of 20 years. Koch et al. [26] argue that the decrease in the daily intake of DnBP by a factor of 2 between two studies performed in 2001/2 and 2007 might be explained by the substitution of DnBP with DiBP that took place in the years between the two studies. This was supported by the authors’ observation that DiBP intake roughly matched the drop in DnBP intake.

### Intake from Exposures in the Indoor Environment

In Langer et al. [80] we presented Spearman correlation coefficients for the various pairings of phthalate diesters in settled dust and metabolite levels measured in the children’s urine. Statistically significant positive correlations were observed between DEP and MEP, DnBP and MnBP, DiBP and MiBP, and BBzP and MBzP. No significant correlation was observed between DEHP in dust from the bedrooms/daycare centers and any of its metabolites in the children’s urine. It is noteworthy that in the current work we found nearly identical correlation coefficients with the same statistical significance between the daily intakes calculated from the urinary metabolite concentrations (*DI_urine_*) and the corresponding day-specific daily intakes estimated from the levels of phthalates in the dust (*DI_indoors_*) (Table 4).

Estimated intakes from exposures in the indoor environment were responsible for a large fraction of the total intakes of DEP and DiBP and contributed meaningfully to the total intake of DnBP. Most of the intake from indoors occurred via dermal absorption. In the case of DEP, estimated intakes from dermal absorption often exceeded total intakes estimated from urine samples. A similar pattern was seen by Guo and Kannan [43]. In their case they suggested that intakes estimated from urinary metabolites may underestimate actual total DEP intakes, or that the dermal absorption coefficient they used to estimate intakes from personal care products may have been too large. In our case, it may also reflect an imperfect estimate of gas-phase concentrations or an overestimated dermal permeability coefficient. Dust ingestion contributed ∼1% or less to the total intake (*DI_urine_*) of these phthalates. This is comparable to the findings of Guo and Kannan [43] and Wormuth et al. [41] for children of 4–10 years, while the latter found a larger DiBP intake from dust ingestion for children below 4 years. Of the total intake, inhalation contributed between 1.6% for DnBP to 12.8% for DEP, which is slightly lower than in Wormuth et al. [41]. However, except for DiBP, these results are consistent with those of Guo and Kannan [43] for children and those of Itoh et al. [42] for adults. A relatively small contribution from inhalation was also reported by Chan and Meek [72], Fromme et al. [75] and Little et al. [54].

The intake fraction that our estimates do not account for (*DI_other_*) includes intakes that occur in other environments than the home or daycare and other routes of exposure, such as diet, mouthing of objects, dermal absorption by contact with dust or personal care products or ingestion of soil and personal care products. This contribution from other environments and pathways was responsible for about 85% of the total intake of DnBP and 50% of the total intake of DiBP. Presumably, a substantial fraction of this is due to diet, mouthing of toys and exposure to personal care products [41],[43],[74],[119],[46],[120], [45],[121]. However, Guo et al. [122] compared daily intakes of phthalates estimated from phthalate levels measured in foodstuffs in China with total intakes estimated from earlier published biomonitoring data. The authors concluded that diet may contribute less than 10% to the total intake of low-molecular-weight phthalates. In a 48 hour fasting study, Koch et al. [64] observed that non-dietary pathways are primarily responsible for exposure to low-molecular-weight phthalates. Some of the other sources of exposure, especially personal care products, also contribute to the total intake of DEP [64]. We suspect that our calculated values for DEP intake from dust ingestion, inhalation and dermal absorption are overestimated. Reasons for this are discussed in the previous paragraph (dermal pathway) and in the subsection “Limitations of the study”.

Indoor environmental exposures contributed very little to the total intake of the high-molecular-weight phthalates. This is of no surprise for DEHP, given the large number of studies indicating that diet is the major route of exposure for this compound [41],[75],[69],[42],[43],[123],[122],[64],[76],[108],[70], [124]. Given DEHP’s relatively high mass fraction in dust [79] and low inferred airborne concentration, it is reasonable that dust ingestion was the strongest contributor (>90%) to the total intake from the three non-diet pathways assessed in this study. An identical result was reported by Little et al. [54]. For the younger children in our study, mouthing of objects may also have contributed to the total intake. Given the comparable properties and similar uses of other high-molecular-weight phthalates such as di(isononyl) phthalate (DiNP), di(isodecyl) phthalate (DiDP) and di(propylheptyl) phthalate (DPHP), similar contributions from the three pathways in the indoor environment to the total intake may be expected for these phthalates. The significant correlation between BBzP in dust and MBzP in urine [80] would suggest that indoor environmental exposures play a meaningful role in BBzP’s total intake. This is somewhat at odds with our current results. However, our results are in line with those of of Wormuth et al. [41], Itoh et al. [42] and Guo and Kannan [43], which found significant BBzP intake from diet, personal care products and sprays.

Most studies estimating dermal exposure to phthalates assume that absorption takes place following topical application of personal care products [125],[7],[45] or direct contact with phthalate containing surfaces [126]. To our knowledge, only a few papers have considered dermal absorption of gas-phase phthalates as a possible source of exposure. Xu et al. [52],[53] estimated the exposure to DEHP from vinyl flooring via inhalation of vapor, inhalation of particles, dermal absorption of DEHP transported to the skin from the gas-phase, and oral ingestion of household dust. Although the primary route of exposure was dust ingestion, the authors concluded that dermal absorption of DEHP deposited on skin can be greater than that taken up through inhalation. Carlstedt et al. [127] found significantly higher urinary levels of MBzP among infants with larger body area and PVC flooring in their bedrooms. Transdermal uptake from air is anticipated to be even more pronounced for low-molecular-weight phthalates, which have a large fraction of their total airborne concentration in the gas phase [51]. Little et al. [54] estimated that almost 60% of a child’s indoor exposure to DnBP (emitted from a single product) occurs via dermal absorption from the gas phase. For all three low-molecular-weight phthalates in our study, by far the largest fraction (>80%) of the total indoor intake was a consequence of dermal absorption from the gas-phase (Table 7).

The permeability coefficients that are used to estimate dermal absorption of these phthalates from the gas phase are based on physical-chemical considerations rather than actual measurements. We anticipate that in the future measurements will be conducted that confirm these calculated permeability coefficients. In the meantime, experimental reassurance regarding the reasonableness of these calculations is provided by studies that have measured the transdermal permeation of DEP and DnBP when present in solutions or creams applied to the surface of human skin. In a 1995 study Hagedorn-Leweke et al. [128] measured a dermal flux of ∼ 10^5^ µg/m^2^/h for DnBP from a propylene glycol/water solution saturated with DnBP. More recently, metabolites of DEP and DnBP have been measured in serum [48] and urine [49] following topical application of a cream containing 2% DEP and 2% DnBP. A flux of 830 µg/m^2^/h was measured for DEP, while a flux of 250 µg/m^2^/h was measured for DnBP. These studies demonstrate that both DEP and DnBP rapidly penetrate the skin. Diffusive transport from the gas phase to skin surface lipids initiates the contact with the skin. Indeed, the transport of DnBP from the gas phase to the skin and subsequent partitioning into skin surface lipids has been inferred from simultaneous measurements of air and handwipe levels of DnBP [51]. In brief, we feel that the available evidence supports the approach that we have used to estimate dermal intakes of gaseous phthalate esters.

As expected, dermal absorption from dust adhered to skin had a negligible effect on the total phthalate intake. The median intake on the day before urine sampling (*DI_dermal_dust_*) was one to three orders of magnitude lower than the intakes by the other three exposure routes. There are however several sources of uncertainty in these estimates. There is limited data in the literature on the amount of dust adhered to skin. Additionally, the dermal uptake rates for dust, adapted from Wormuth et al. [41], were based on the uptake rates for cosmetics directly applied onto skin, corrected with a factor of 0.15 to account for the matrix effect. We were unable to find specific data for each phthalate and the same matrix value was applied for all target phthalates. Further discussion of the contribution to the total phthalate intake of dust adhered to skin is presented in part C of Discussion S1.

### Comparison with Tolerable Daily Intake (TDI) Values

Assuming that DiBP and DnBP have similar TDIs, the total DiBP intake of 23 children exceeded the putative TDI. This was the highest number of exceedences among the five phthalates, followed by 22 children, whose total DnBP intake exceeded its respective TDI. The median intakes of DnBP, DiBP and DEHP corresponded to 33, 29 and 9% of their respective TDIs, comparable to the values of 19%, 21% and 8.9% reported by Koch et al. [26] and 53% for the sum of DnBP and DiBP, and 11% for DEHP calculated from median intake values for children of age 6–10 years, reported by Frederiksen et al. [113]. Although the daily intake of DEHP calculated from the urinary concentrations was the highest among the five phthalates, only 3 total intake values exceeded the TDI for DEHP. The US EPA’s Reference Dose for DEHP is based on increased relative liver weight instead of reproductive and developmental effects [98], and its value was exceeded by 16 children.

Somewhat surprisingly, 14 children exceeded the TDI of DiBP, based *only* on exposures that occurred in indoor environments. These children were not necessarily the same children whose total intake calculated from their urinary levels exceeded the TDI. 11 out of the 14 children had an intake via dermal absorption alone (*WI_dermal_gas/_7*) exceeding the TDI. It is important to note, that the current TDI values are based on dietary or total intake of phthalates and are most appropriate for dietary exposure. There are no limit values for phthalate intake via other pathways. However, the nature of the exposure pathway may have an impact on the health effect of a contaminant. While ingested compounds pass through the intestines and liver before entering the blood stream, exposure via inhalation may impact the respiratory system first. Transdermal exposure may impact the skin and can lead to systemic effects by direct delivery of the contaminant to organs via blood. Taken together, our intake estimates indicating that transdermal exposure to DiBP may exceed tolerable daily intake levels further support the potential significance of exposure to phthalates in the indoor environment. These findings should be confirmed and differences in health effects resulting from intakes via different exposure pathways should be further investigated.

Applying the approach of a cumulative tolerable daily intake, as recently introduced by Koch et al. [26], to the three phthalates for which TDI values are available based on the same health endpoint (effects on reproduction and development), we found that 30% of children exceeded the *TDI_cum_*. This agrees with the results of Koch et al. [26], who found exceedences of *TDI_cum_* in 25% of their study population. Søeborg et al. [40] found 19 out of 129 (15%) Danish children and adolescents between 6 and 21 years of age exceeding the cumulative tolerable daily intake for these three phthalates. In our study the cumulative intake of the three phthalates reached ∼80% of *TDI_cum_* at the median level and ∼220% at the 95^th^ percentile level, compared to 56% and 183%, respectively, reported by Koch et al. [26] and 51% and 129%, respectively, reported by Søeborg et al. [40] for older children and adolescents. Calculating and summing the individual portions of TDI reached by the median and 95^th^ percentile intakes of DnBP+DiBP and DEHP as reported in Frederiksen et al. [113] for children aged 6–10 years, 64% and 235% of TDI_cum_ would be reached, respectively. Although this latter approach is imperfect, these results reflect somewhat similar exposures of German and Danish children to phthalates. The relatively high fraction of children exceeding the *TDI_cum_* is noteworthy. The fact that 26 children (6%) exceeded the *TDI_cum_* simply due to dust ingestion, inhalation and dermal absorption of gas-phase phthalates, supports the hypothesis that exposure to phthalates in the indoor environment can substantially contribute to the total phthalate exposure.

Other common indoor pollutants are suspected endocrine disruptors with anti-androgenic effects similar to those attributed to DnBP, DiBP and DEHP. These include BBzP, diisononyl phthalate (DINP), bisphenol A, selected polychlorinated biphenyls (PCBs), selected polybrominated diphenyl ethers (PBDEs) and pesticides such as vinclozolin, procymidone [129],[36],[30],[33],[34],[130],[131]. As their anti-androgenic properties are confirmed and reliable TDI values become established, intakes of these compounds should be included when calculating a summed intake to be compared to *TDI_cum_*. The inclusion of additional indoor pollutants in such an assessment is expected to increase the fraction of children whose net daily intake of endocrine disrupting chemicals exceeds an established cumulative TDI.

### Limitations of the Study

To our knowledge, this is the largest study that compares children’s phthalate intakes estimated from urinary phthalate metabolite concentrations with intakes estimated from dust ingestion, inhalation and dermal absorption. Moreover, this may be the first study that performs these calculations on a child-by-child basis while taking into account the children’s exposures in both their home and daycare environments. Nevertheless, it is important to recognize that our estimates of daily phthalate intakes from the indoor environment required numerous assumptions and approximations.

In the case of DnBP, DiBP, BBzP and DEHP, the ratio (*C_dust_/C_g_*) was calculated using equation (8) and the appropriate K_oa_ for the phthalate in question. K_oa_ was also used to calculate each phthalate’s partitioning between the gas-phase and airborne particles (equation (10)). Equation (8) is based on a limited number of studies [56]. Additionally, values of K_oa_ have not been measured for these phthalates and have instead been estimated using a structure-activity relationship (SPARC v4.6). These K_oa_ values could be off by an order of magnitude or more [132]. Moreover, to estimate the total airborne phthalate concentrations (*C_g_+C_p_*), we assumed that all homes and daycare centers had the same average indoor concentration of airborne particles (20 µg/m^3^), and that these airborne particles had the same density and volume fraction of organic matter. In actuality, each of these parameters likely varied among the different indoor environments. Of the phthalates targeted in this study, BBzP is the most sensitive to errors in K_oa_ and, to a lesser extent, in assumptions regarding airborne particles. It is also the targeted phthalate for which there appears to be no single dominant indoor pathway. An inaccurate estimate of K_oa_ for BBzP may be partially responsible for what appear to be underestimated contributions from its indoor pathways.

An additional error may stem from the limited accuracy of phthalate metabolite concentrations in urine. We compared metabolite concentrations determined by two different analytical procedures for a subset of our urine samples [80]. The average difference between the two methods was 30% for the geometric mean concentrations of eight metabolites. In contrast, we judge the analytical errors in the phthalate mass fractions in the dust to be below 10%.

Several of the exposure input parameters are poorly characterized and some (e.g., inhalation rate, dust ingestion rate, fraction of time spent in different environments) are anticipated to vary among the children and over time. The dermal permeability coefficients are based on the best available, but often limited, knowledge [51]. Additionally, these coefficients do not account for the fact that the skin of children may be more permeable than the skin of adults [96].

The number of controlled studies on the metabolism of phthalates in humans is small [87], and different urinary excretion factors (F_ue_) are reported in the literature. For some of the phthalates, F_ue_’s have been obtained from studies with only a few adult volunteers [89],[133]. Furthermore, several papers suggest that metabolism in children differs from that in adults [112],[85],[110]. In the present study, we have used a urinary excretion factor of 0.69 for DEP, DnBP and DiBP. A urinary excretion factor has yet to be measured for DEP/MEP. After we had completed our analysis, Koch et al. [101] reported a measured urinary excretion factor of 0.71 for DiBP/MiBP – close to the value that we used.

Comparisons of total intakes calculated from urinary metabolite levels with intakes estimated from various exposure pathways, including the calculation of *DI_other_* from equation (6), assume that the fraction of a given phthalate excreted in urine is identical for all exposure pathways. However, the urinary excretion factors have been determined from excretion over a short time period after controlled *oral* administration. Whether the fraction excreted after inhalation or dermal absorption is the same as that following ingestion is currently not known.

Both DnBP and BBzP metabolize to form MnBP. In our calculations we assumed that all MnBP was derived from DnBP. However, we do not anticipate this fact to be a source of significant error. According to Anderson et al. [133], less than 10% of BBzP metabolizes to MnBP in humans.

The association between intakes estimated from the urinary metabolite concentrations and intakes from dust mass fractions may be influenced by the relatively long time (up to a year) that elapsed between dust collection in the homes and daycare facilities and urine sample collection. Moreover, the mass fractions of phthalates in dust correspond to exposures over an extended period of time, while levels of phthalate metabolites in urine correspond to exposures that occurred over the previous 24 to 36 hours. Certain short-term conditions that we could not account for (e.g. intake of phthalate-containing drugs [134],[135], foods or beverages), may have influenced our intake estimates derived from urine measurements. In spite of potentially large within-person daily variability of urinary phthalate metabolites, some authors have suggested that a single sample may reasonably predict average metabolite concentrations over a period of several months [136],[137],[138],[5].

Frederiksen et al. [113] observed substantially higher metabolite levels in first morning urine samples compared to corresponding 24-hour urine samples. On the other hand, temporal variations in metabolite concentrations in the urine during the course of a day may differ between various monoesters, possibly reflecting different exposures at different times of the day [111],[139],[123].

When estimating the contribution of inhalation to the total intake, we assumed 100% retention and 100% absorption. This may overestimate phthalate intake via the inhalation pathway. Hawley et al. [96] assumed that 75% of inhaled particulate matter is retained and 100% of the contaminant in inhaled dust is absorbed. Wormuth et al. [41] assumed an overall uptake rate for inhalation of 75% for adults and 100% for children. Jakubowski and Czerczak [140] summarized experimental studies that determined the rates at which various volatile organic compounds (VOCs) present in inhaled air are retained in the lung. The lung retention rates of the studied VOCs varied between 20–90%, and, for certain compounds, changed with the duration of exposure. Similarly, the arterial blood concentration resulting from the inhalation of a phthalate is expected to depend on its gaseous concentration, the duration of exposure, and its physicochemical properties [141].

Regardless of these limitations, we believe that the present study provides valuable insights regarding the relative contribution of the indoor environment to the total phthalate exposure of Danish children. Analyses of the relationship between daily phthalate intakes in the present study and the children’s health status are ongoing.

### Conclusions

Total phthalate intakes calculated from metabolite levels in the urine were comparable to those reported in recent studies conducted in Denmark and Germany. Children’s exposure to phthalates occurring indoors via dust ingestion, inhalation and dermal absorption can meaningfully contribute to the total intake of the low-molecular-weight phthalates such as DEP, DnBP and DiBP. Dermal absorption and inhalation appear to be the most important routes of environmental exposure for these chemicals. Exposures occurring in indoor environments contributed only a small fraction of the total intake for DEHP. Most of its exposure attributable to the indoor environment occurred through dust ingestion. However, less than 10% of its total daily intake came via this pathway.

For 22 children, the total intake of DnBP from all sources and exposure pathways exceeded its TDI. In the case of DiBP, the total intake of 23 children exceeded its putative TDI (assumed to be the same as that of DnBP). Even when only exposures that occurred in the home and daycare environments were considered, a number of children had DiBP intakes that exceeded the TDI. This may reflect an increased substitution of DiBP for other phthalates found in indoor environments [63],[26],[109],[118], [67],[66]. Nearly every third child had a summed intake of DnBP, DiBP and DEHP that exceeded the cumulative TDI of 100% for these compounds. Considering exclusively exposures that occurred in the indoor environment (daily indoor intakes based on one-week averages (*WI_indoors/_7*)), 6% of the children had summed intakes of DnBP, DiBP and DEHP that exceeded the cumulative TDI for these three phthalates. Assuming that the metabolite levels in 24-hour urine samples could be lower than in our first morning urines [113], our current daily intakes estimated from the environmental exposure in the home and daycare would constitute an even larger fraction of the total intake. Taken together, the indoor environment appears to be an important source of phthalate exposure.

To better characterize exposure, further research is needed regarding the absorption, distribution and elimination of phthalates via different pathways, as well as the role of the exposure pathway in determining various adverse health effects. Additionally, future research should address the health effects of simultaneous human exposure to multiple phthalates and other potential endocrine disruptors.

## Supporting Information

Figure S1 **Cumulative frequency distributions of total phthalate intakes.** Distributions are shown for total intakes calculated from the metabolite concentrations measured in urine (*DI_urine_*) and estimated from the children’s exposure to phthalates in the indoor environment (sum of dust ingestion, inhalation and dermal absorption). The latter is depicted both as the daily intake on the day before urine sampling (*DI_indoors_*) and as the average daily intake from a week-long exposure (*WI_indoors/_*7): A) DEP, B) DnBP, C) DiBP, D) BBzP and E) DEHP. The solid horizontal line indicates the TDI value. The TDI value for BBzP (500 µg/d/kg-bw) is not indicated in the plot.
(TIF)

Table S1 **Mass-fractions (µg/g) of phthalates in dust samples collected from homes and daycare centers and concentrations (ng/mL) of the phthalate metabolites in urine samples.**
(DOCX)

Table S2 **Contribution (%) of the home and daycare environments to the weekly intake from each pathway and to the total weekly intake (** ***WI_indoors_*** **).**
(DOCX)

Table S3 **Daily phthalate intakes (µg/d/kg-bw) of children estimated indirectly from exposure to various media (food, air, water, soil, dust).**
(DOCX)

Discussion S1 **Further discussion on relevant issues.** A. Discussion on the daily phthalate intakes estimated indirectly from models based on phthalate levels in various media, B. Discussion on potential bias resulting from the case-base selection of the study population, C. Discussion on the intake from dermal absorption from dust adhered to skin.
(DOCX)
