# Supplementary figures and images for: Children’s Phthalate Intakes and Resultant Cumulative Exposures Estimated from Urine Compared with Estimates from Dust Ingestion, Inhalation and Dermal Absorption in Their Homes and Daycare Centers
Source: PLoS One. 2013 Apr 23;8(4):e62442. doi: 10.1371/journal.pone.0062442 (PMC3633888; doi:10.1371/journal.pone.0062442)

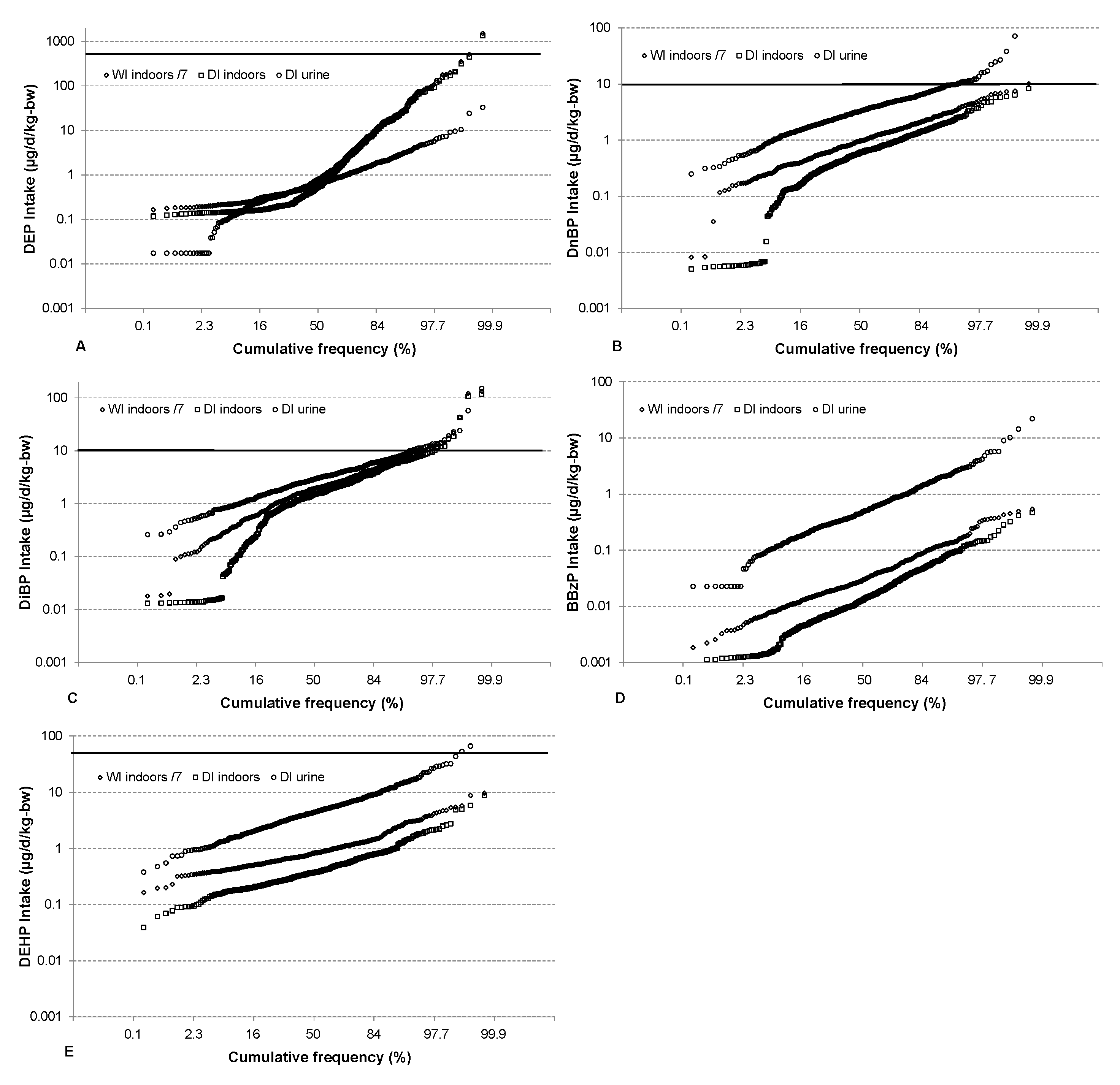

Supplement: Figure S1 — Cumulative frequency distributions of total phthalate intakes. Distributions are shown for total intakes calculated from the metabolite concentrations measured in urine (DIurine) and estimated from the children’s exposure to phthalates in the indoor environment (sum of dust ingestion, inhalation and dermal absorption). The latter is depicted both as the daily intake on the day before urine sampling (DIindoors) and as the average daily intake from a week-long exposure (WIindoors/7): A) DEP, B) DnBP, C) DiBP, D) BBzP and E) DEHP. The solid horizontal line indicates the TDI value. The TDI value for BBzP (500 µg/d/kg-bw) is not indicated in the plot. (TIF) [file pone.0062442.s001.tif]
