# Supplementary material for: Children’s Phthalate Intakes and Resultant Cumulative Exposures Estimated from Urine Compared with Estimates from Dust Ingestion, Inhalation and Dermal Absorption in Their Homes and Daycare Centers
Source: PLoS One. 2013 Apr 23;8(4):e62442. doi: 10.1371/journal.pone.0062442 (PMC3633888; doi:10.1371/journal.pone.0062442)
Supplement: Table S1 — Mass-fractions (µg/g) of phthalates in dust samples collected from homes and daycare centers and concentrations (ng/mL) of the phthalate metabolites in urine samples. (DOCX) [file pone.0062442.s002.docx]

**Children’s Phthalate Intakes and Resultant Cumulative Exposures Estimated from Urine Compared with Estimates from Dust Ingestion, Inhalation and Dermal Absorption in their Homes and Daycare Centers**

**Supporting Information**

**Table S1.** Mass-fractions (µg/g) of phthalates in dust samples collected from homes and daycare centers and concentrations (ng/mL) of the phthalate metabolites in urine samples.

|  | Min. | Max. | Median |
| --- | --- | --- | --- |
| **Phthalate dust mass fractions – Homes (n=497)** [1]  DEP  DnBP  DiBP  BBzP  DEHP | 0.35  0.18  0.26  0.7  12.7 | 6194  253  2496  285  6611 | 1.7  15  27  3.7  210 |
| **Phthalate dust mass fractions – Daycare centers (n=151)** [1]  DEP  DnBP  DiBP  BBzP  DEHP | 0.35  0.18  0.26  0.7  101 | 19.5  440  652  293  5566 | 2.2  38  23  17  500 |
| **Phthalate metabolite in urine (n=441)** [2]  MEP  MnBP  MiBP  MBzP  MEHP  MEHHP  MEOHP  MECPP | 0.47  6.15  6.45  0.61  0.14  0.53  2.87  3.18 | 888  4007  3750  597  530  2580  2092  5541 | 16.6  80.1  72.2  13.0  4.7  33.2  17.6  34.5 |

**References**

1. Langer S, Weschler CJ, Fischer A, Bekö G, Toftum J, et al. (2010) Phthalate and PAH concentrations in dust collected from Danish homes and daycare centers. Atmospheric Environment 44: 2294-2301.

2. Langer S, Weschler CJ, Bekö G, Toftum J, Brive L, et al. (2013) Phthalate metabolites in urine samples of danish children and correlations with phthalates in dust samples from their homes and daycare centers. International Journal of Hygiene and Environmental Health: Submitted.
