# Supplementary material for: Children’s Phthalate Intakes and Resultant Cumulative Exposures Estimated from Urine Compared with Estimates from Dust Ingestion, Inhalation and Dermal Absorption in Their Homes and Daycare Centers
Source: PLoS One. 2013 Apr 23;8(4):e62442. doi: 10.1371/journal.pone.0062442 (PMC3633888; doi:10.1371/journal.pone.0062442)
Supplement: Table S2 — Contribution (%) of the home and daycare environments to the weekly intake from each pathway and to the total weekly intake ( WIindoors ). (DOCX) [file pone.0062442.s003.docx]

**Children’s Phthalate Intakes and Resultant Cumulative Exposures Estimated from Urine Compared with Estimates from Dust Ingestion, Inhalation and Dermal Absorption in their Homes and Daycare Centers**

**Supporting Information**

**Table S2**. Contribution (%) of the home and daycare environments to the weekly intake from each pathway and to the total weekly intake (*WI_indoors_*).

|  |  | DEP | DnBP | DiBP | BBzP | DEHP |
| --- | --- | --- | --- | --- | --- | --- |
| *WI_ingest_dust_* from: | Home, P50(Mean) | 61.0(59.3) | 37.7(39.5) | 67.3(59.3) | 28.9(37.1) | 38.9(41.0) |
|  | DCC, P50(Mean) | 39.0(40.7) | 62.3(60.5) | 32.7(40.7) | 71.1(62.9) | 61.1(59.0) |
| *WI_inhalation_* from: | Home, P50(Mean) | 75.0(70.8) | 53.6(49.5) | 79.7(68.3) | 43.7(46.8) | 55.0(54.2) |
|  | DCC, P50(Mean) | 25.0(29.2) | 46.4(50.5) | 20.3(31.7) | 56.3(53.2) | 45.0(45.8) |
| *WI_dermal_gas_* from: | Home, P50(Mean) | 78.6(73.6) | 58.6(52.5) | 82.8(70.7) | 48.7(49.9) | 59.9(58.3) |
|  | DCC, P50(Mean) | 21.4(26.4) | 41.4(47.5) | 17.2(29.3) | 51.3(50.1) | 40.1(41.7) |
| *WI_indoors_* from: | Home, P50(Mean) | 78.0(73.1) | 56.9(51.4) | 81.9(70.1) | 33.2(40.0) | 40.0(41.9) |
|  | DCC, P50(Mean) | 22.0(26.9) | 43.1(48.6) | 18.1(29.9) | 66.8(60.0) | 60.0(58.1) |
