# Supplementary material for: Children’s Phthalate Intakes and Resultant Cumulative Exposures Estimated from Urine Compared with Estimates from Dust Ingestion, Inhalation and Dermal Absorption in Their Homes and Daycare Centers
Source: PLoS One. 2013 Apr 23;8(4):e62442. doi: 10.1371/journal.pone.0062442 (PMC3633888; doi:10.1371/journal.pone.0062442)
Supplement: Table S3 — Daily phthalate intakes (µg/d/kg-bw) of children estimated indirectly from exposure to various media (food, air, water, soil, dust). (DOCX) [file pone.0062442.s004.docx]

**Children’s Phthalate Intakes and Resultant Cumulative Exposures Estimated from Urine Compared with Estimates from Dust Ingestion, Inhalation and Dermal Absorption in their Homes and Daycare Centers**

**Supporting Information**

**Table S3.** Daily phthalate intakes (µg/d/kg-bw) of children estimated indirectly from exposure to various media (food, air, water, soil, dust).

| **Reference/age** | **Survey years** | **Country** | **n** | **DEP** | **DnBP** | **DiBP** | **BBzP** | **DEHP** |
| --- | --- | --- | --- | --- | --- | --- | --- | --- |
| Wormuth et al. [1] (1-3 yrs)^c^ | - | EU | - | 1.49^b^ | 2.55^b^ | 0.68^b^ | 0.31^b^ | 6.31^b^ |
| Wormuth et al. [1] (4-10 yrs)^c^ | - | EU | - | 0.76^b^ | 1.23^b^ | 0.29^b^ | 0.06^b^ | 1.97^b^ |
| Fromme et al. [2] (0-4 yrs) | - | Germany | - | 0.61^d^ | 4.92^d^ | - | 0.68^d^ | 24.0^d^ |
| Meek and Chan; Chan and Meek [3,4] (0.5-4 yrs) | - | Canada | - | - | 5.0^e^ | - | - | 19^e^ |
| Meek and Chan; Chan and Meek [3,4] (5-11 yrs) | - | Canada | - | - | 4.3^e^ | - | - | 14^e^ |
| Clark et al. [5] (5-11 yrs) | - | USA, Canada | - | 5.7^f^ | 11^f^ - | | 7.9^f^ | 18.9^f^ |
| Clark et al. [5] (0.5-4 yrs) | - | USA, Canada | - | 10.6^f^ | 14f - | | 9.3^f^ | 25.8^f^ |
| Clark et al. [6] (5-11 yrs) | - | USA, Canada | - | 0.93^f^ | 2.4^f^ | 2.1^f^ | 0.97^f^ | 20^f^ |
| Clark et al. [6] (0.5-4 yrs) | - | USA, Canada | - | 1.2^f^ | 3.4^f^ | 2.6^f^ | 1.5^f^ | 30^f^ |
| Bosgra et al. [7] (1 yr) | - | - | - | - | - | - | - | 13.2^a,g^ |

| **This study, (3-6 yrs)^h^** | **08-09** | **Denmark** | **431** | **0.62** | **3.26** | **2.93** | **0.49** | **4.42** |
| --- | --- | --- | --- | --- | --- | --- | --- | --- |

All values are medians except when indicated otherwise.

^a^ Geometric Mean

^b^ Mean value

^c^ Exact values from Heudorf et al. [8]

^d^ DEP and BBzP from indoor air and household dust, DnBP and DEHP from indoor air, household dust and food

^e^ from ambient air, indoor air, food, drinking water, soil

^f^ from food, air, water, soil and dust

^g^ from food, indoor air and toys

^h^ from urinary phthalate metabolite concentrations

**References**

1. Wormuth M, Scheringer M, Vollenweider M, Hungerbuhler K. (2006) What are the sources of exposure to eight frequently used phthalic acid esters in europeans? Risk Analysis 26: 803-824.

2. Fromme H, Lahrz T, Piloty M, Gebhart H, Oddoy A, et al. (2004) Occurrence of phthalates and musk fragrances in indoor air and dust from apartments and kindergartens in berlin (Germany). Indoor Air 14: 188-195.

3. Meek ME, Chan PKL. (1994) Bis(2-ethylhexyl)phthalate - evaluation of risks to health from environmental exposure in Canada. Environmental Carcinogenesis & Ecotoxicology Reviews-Part C of Journal of Environmental Science and Health 12: 179-194.

4. Chan PKL, Meek ME. (1994) Di-N-butyl phthalate - evaluation of risks to health from environmental exposure in Canada. Environmental Carcinogenesis & Ecotoxicology Reviews-Part C of Journal of Environmental Science and Health 12: 257-268.

5. Clark K, Cousins I, MacKay D. (2003) Assessment of critical exposure pathways. In: Staples CA, editor. The Handbook of Environmental Chemistry, 3Q. Phthalate Esters. New York: Springer. pp. 227-262.

6. Clark KE, David RM, Guinn R, Kramarz KW, Lampi MA, et al. (2011) Modeling human exposure to phthalate esters: A comparison of indirect and biomonitoring estimation methods. Human and Ecological Risk Assessment 17: 923-965.

7. Bosgra S, Bos PMJ, Vermeire TG, Luit RJ, Slob W. (2005) Probabilistic risk characterization: An example with di(2-ethylhexyl) phthalate. Regulatory Toxicology and Pharmacology 43: 104-113.

8. Heudorf U, Mersch-Sundermann V, Angerer J. (2007) Phthalates: Toxicology and exposure. International Journal of Hygiene and Environmental Health 210: 623-634.
