# Supplementary material for: Children’s Phthalate Intakes and Resultant Cumulative Exposures Estimated from Urine Compared with Estimates from Dust Ingestion, Inhalation and Dermal Absorption in Their Homes and Daycare Centers
Source: PLoS One. 2013 Apr 23;8(4):e62442. doi: 10.1371/journal.pone.0062442 (PMC3633888; doi:10.1371/journal.pone.0062442)
Supplement: Discussion S1 — Further discussion on relevant issues. A. Discussion on the daily phthalate intakes estimated indirectly from models based on phthalate levels in various media, B. Discussion on potential bias resulting from the case-base selection of the study population, C. Discussion on the intake from dermal absorption from dust adhered to skin. (DOCX) [file pone.0062442.s005.docx]

**Children’s Phthalate Intakes and Resultant Cumulative Exposures Estimated from Urine Compared with Estimates from Dust Ingestion, Inhalation and Dermal Absorption in their Homes and Daycare Centers**

**Supporting Information**

**Discussion S1. Further discussion on relevant issues.** A. Discussion on the daily phthalate intakes estimated indirectly from models based on phthalate levels in various media, B. Discussion on potential bias resulting from the case-base selection of the study population, C. Discussion on the intake from dermal absorption from dust adhered to skin.

**A. Discussion on the** **daily phthalate intakes estimated indirectly from models based on phthalate levels in various media**

Intakes of DEHP determined indirectly from exposure models were mostly higher than our estimates based on urinary metabolite concentrations (Table S3). Similar tendencies were observed in some of the models for DEP, DnBP and BBzP. The results of Wormuth et al. [1] and Clark et al. [2] were, however, in a reasonably good agreement with our current intake estimates for most of the phthalates. Exposure models rely on surveys of product use, measurements or estimates of phthalate concentrations in various media, estimates on potential sources of exposure to those media, as well as on various pharmacokinetic assumptions. Such data may not be up-to-date at the time of a given study [3]. Obtaining reliable phthalate concentrations in various exposure media can be problematic [4].

**B. Discussion on potential bias resulting from the case-base selection of the study population**

We believe that only a small bias was introduced by the case-base selection of the study population. The mass fractions of phthalates in the dust collected from the homes and daycare centers were similar for the two groups. The average base-to-case ratio of dust mass fractions for all phthalates in both homes and daycares was 1.09 (between 0.85 and 1.48). The largest relative difference between the two groups was observed for DnBP in the homes, with geometric means of 6.4µg/g for the cases and 9.5µg/g for the bases (not statistically significant by Student’s two sample t-test and Wilcoxon rank-sum test). The concentrations of phthalate metabolites in the urine were somewhat higher for the base group than for the case group. The average base-to-case ratio of phthalates metabolites was 1.11 (between 1.02 and 1.16). The largest relative difference was observed for MnBP (geometric mean: 71.4 ng/ml for the cases and 82.8 ng/ml for the bases). Again, the difference was not statistically significant. Indeed, these tendencies were reflected in the results of the intake calculations. There were no differences between the two groups in the daily intakes calculated either from phthalate metabolite levels in the urine or from phthalate mass fractions in the dust. More bases than cases exceeded the cumulative tolerable daily intake, based on the total daily phthalate intake calculated from the urinary metabolite levels (84 vs. 47; 18 vs. 4 for the individual TDI of DnBP; 11 vs. 12 for DiBP). On the contrary, 10 bases and 16 cases had a cumulative TDI above 100% from a week-long exposure via three pathways in the indoor environment (4 vs. 10 for the individual TDI of DiBP).

**C. Discussion on the intake from dermal absorption from dust adhered to skin**

Dermal exposure from dust adhered to skin made a negligible contribution to total phthalate intake. The median intake on the day before urine sampling (*DI_dermal_dust_*) was between 0.00007 – 0.0006 µg/d/kg-bw. This is in the same order of magnitude as reported by Guo and Kannan [5] and one to three orders of magnitude lower than the intakes by the other three exposure routes. Kang et al. [6] reported several orders of magnitude higher intakes of DBP and DEHP through dermal contact with indoor dust in China. There are however several sources of uncertainty in these estimates. There is limited amount of data in the literature on the amount of dust adhered to skin. The US EPA [7],[8] summarizes the work that has been done on adherence of solids to skin. Most of these studies investigated the adherence of soil to skin during specific few-hour activities both indoors and outdoors. Other studies used the gravimetric method after direct application of soils onto skin. The experiments were performed with small sample sizes. The mass of particles adhered to skin in milligrams per cm^2^ of skin surface, estimated from these experiments, is considered in the current study as a constant dust mass in contact with skin, with the dust mass containing a constant mass fraction of phthalates. This approach may not match reality. Moreover, dust deposition on skin may vary with behavior, clothing, duration of activity, season, dust levels and size-distribution of the dust. Additionally, the dermal uptake rates for dust, which we adapted from Wormuth et al. [1], were based on uptake rates for cosmetics directly applied to skin, corrected with a factor of 0.15 to account for the matrix effect that reduces the absorption rate from soil and dust. The matrix effect is compound-specific [9]. We were unable to find specific data for each phthalate and the same matrix value was used for all target phthalates.

For reasons outlined above and due to the very low concentrations of phthalates in soil, we did not include the potential intake from soil adhered to skin in our analyses. Similarly, due to low phthalate concentrations in outdoor air coupled with the small contribution of inhalation to the total intake, we did not consider outdoor exposures to airborne phthalates in our analyses [10],[11],[3],[12],[13],[14].

**References**

1. Wormuth M, Scheringer M, Vollenweider M, Hungerbuhler K. (2006) What are the sources of exposure to eight frequently used phthalic acid esters in Europeans? Risk Analysis 26: 803-824.

2. Clark KE, David RM, Guinn R, Kramarz KW, Lampi MA, et al. (2011) Modeling human exposure to phthalate esters: A comparison of indirect and biomonitoring estimation methods. Human and Ecological Risk Assessment 17: 923-965.

3. Clark K, Cousins I, MacKay D. (2003) Assessment of critical exposure pathways. In: Staples CA, editor. The Handbook of Environmental Chemistry, 3Q. Phthalate Esters. New York: Springer. pp. 227-262.

4. Franco A, Prevedouros K, Alli R, Cousins IT. (2007) Comparison and analysis of different approaches for estimating the human exposure to phthalate esters. Environment International 33: 283-291.

5. Guo Y, Kannan K. (2011) Comparative assessment of human exposure to phthalate esters from house dust in china and the united states. Environmental Science & Technology 45: 3788-3794.

6. Kang Y, Man YB, Cheung KC, Wong MH. (2012) Risk assessment of human exposure to bioaccessible phthalate esters via indoor dust around the pearl river delta. Environmental Science & Technology 46: 8422-8430.

7. US EPA. (2009) Child-specific exposure factors handbook. Available: http://cfpub.epa.gov/ncea/cfm/recordisplay.cfm?deid=199243. Accessed 17 December 2012.

8. US EPA. (1997) Exposure factors handbook. Available: http://epa.gov/oppt/exposure/pubs/usepa_1997_efh.pdf. Accessed 17 December 2012.

9. Hawley JK. (1985) Assessment of health risk from exposure to contaminated soil. Risk Analysis 5: 289-302.

10. Wang XK, Guo WL, Meng PR, Gan JA. (2002) Analysis of phthalate esters in air, soil and plants in plastic film greenhouse. Chinese Chemical Letters 13: 557-560.

11. Ma LL, Chu SG, Xu XB. (2003) Phthalate residues in greenhouse soil from Beijing suburbs, people's republic of china. Bulletin of Environmental Contamination and Toxicology 71: 394-399.

12. Teil MJ, Blanchard M, Chevreuil M. (2006) Atmospheric fate of phthalate esters in an urban area (Paris-France). Science of the Total Environment 354: 212-223.

13. Wang P, Wang SL, Fan CQ. (2008) Atmospheric distribution of particulate- and gas-phase phthalic esters (PAEs) in a metropolitan city, Nanjing, east China. Chemosphere 72: 1567-1572.

14. Rudel RA, Dodson RE, Perovich LJ, Morello-Frosch R, Camann DE, et al. (2010) Semivolatile endocrine-disrupting compounds in paired indoor and outdoor air in two northern California communities. Environmental Science & Technology 44: 6583-6590.
